# A φSa3int (NM3) Prophage Domestication in *Staphylococcus aureus* Leads to Increased Virulence Through Human Immune Evasion

**DOI:** 10.1002/mco2.70313

**Published:** 2025-08-08

**Authors:** Roshan Nepal, Ghais Houtak, George Bouras, Sholeh Feizi, Gohar Shaghayegh, Keith Shearwin, Mahnaz Ramezanpour, Alkis James Psaltis, Peter‐John Wormald, Sarah Vreugde

**Affiliations:** ^1^ The Faculty of Health and Medical Sciences The University of Adelaide Adelaide Australia; ^2^ The Department of Surgery‐Otolaryngology Head and Neck Surgery The Basil Hetzel Institute for Translational Health Research Central Adelaide Local Health Network Adelaide South Australia Australia; ^3^ Commonwealth Scientific and Industrial Research Organisation (CSIRO) Agriculture and Food Livestock and Aquaculture Hobart TAS Australia; ^4^ School of Biological Sciences Faculty of Sciences Engineering and Technology The University of Adelaide Adelaide Australia

**Keywords:** bacteriophage, chronic rhinosinusitis, microbe–host interaction, NM3 prophage, phage, Sa3int prophage

## Abstract

*Staphylococcus aureus* with varying virulence is often isolated from chronic rhinosinusitis (CRS) patients and impacts disease severity. Prophage‐mediated virulence, particularly encoded by φSa3int (NM3) prophages, which often encodes human immune‐evasion cluster genes is well known, but how a new prophage domestication impacts overall expression of core bacterial genes, and the expression of resident prophages is understudied. To understand this, we transduced a φSa3int prophage recovered from hyper‐biofilm forming mucoid *S. aureus* (SA333) into a high‐biofilm forming non‐mucoid *S. aureus* (SA222) recovered from same CRS patient but at different time points. Upon φSa3int prophage domestication, we observed a significant upregulation of 21 exoproteins including human immune‐evasion toxins and an intercellular adhesion protein B (IcaB). Further, φSa3int prophage domestication led to reduced phagocytosis implying φSa3int prophage mediates escape of *S. aureus* from human innate immunity. Our data further show that in addition to adding novel prophage‐encoded virulence, φSa3int prophage domestication also affects the expression of non‐prophage (bacterial) genes and suppresses expression of structural proteins of resident prophages. Since strains without prophage or with specific prophages have varying virulence and pathogenicity, targeted identification virulence factors associated with mobile genetic elements (MGEs) in addition to species identification may lead to better personalized therapy, particularly in chronic infections.

## Graphical abstract

Prophages play a significant role in modulating bacterial virulence and pathogenicity. Our results suggest domestication of φSa3int prophage leads to reduced phagocytosis mediating escape of *S. aureus* from human innate immunity. As such, targeted identification of mobile genetic elements (MGEs) in addition to species and strain identification may lead to better‐personalized therapy as not all bacteria carry the same MGEs

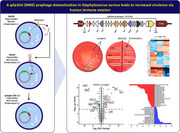

## 1 Introduction

Bacteria often harbor dormant phage DNA, also known as prophages, embedded within their chromosomes. These prophages can confer auxiliary functions, frequently increasing bacterial fitness. However, prophages can also carry various virulence factors (VFs), toxins, and antimicrobial resistance genes (ARGs), such that lysogens (bacteria carrying a prophage) are often considered more virulent than the corresponding prophage‐free strain [1, 2]. Prophages can be induced spontaneously or in response to various extrinsic factors like UV, sub‐lethal antibiotics, and chemicals [3]. Although spontaneous prophage induction (SPI) usually occurs at a low level and kills a small fraction of bacterial cells within a population, the released phages can infect and lysogenize other susceptible strains in the niche, thereby transducing the prophage‐encoded VFs (PVFs) horizontally. Further, the induction of prophages into phage particles can also activate an antiviral immune response in mammalian cells that protects bacteria from phagocytosis [4, 5]. It is well established that prophage induction is enhanced under sub‐lethal concentrations of various antibiotics and chemicals [6, 7, 8]. Moreover, some studies have also found that prophage domestication and induction enhance biofilm formation, further increasing bacterial survival and fitness [9, 10]. As bacteria often acquire virulent traits under inadequate or inappropriate antimicrobial treatment regimens despite the DNA replication cost involved, it is essential to understand prophage diversity, prophage dispersal, and their role in virulence dissemination. Evolutionary studies suggest that many bacterial infections are dominated by a small number of coevolved clones that are virulent and pathogenic compared with their commensal counterpart [11, 12]. Therefore, understanding the dynamics of prophage‐mediated virulence dissemination may provide information about the origin and spread of virulent clones.

*Staphylococcus aureus (S. aureus)* is a genetically and metabolically diverse, highly successful opportunistic bacterial pathogen colonizing the mucosal surfaces of approximately 30% of humans [13]*. S. aureus* is often isolated from the sinuses of chronic rhinosinusitis (CRS) patients, more often in CRS with nasal polyposis (CRSwNP) compared with CRS without nasal polyposis (CRSsNP) and is thought to play role in severity of chronic infection of the sinuses [14]. However, our previous studies involving core genome analysis of *S. aureus* recovered from CRSwNP and CRSsNP did not find significant difference in core‐genome virulence and ARGs [15]. Lately, research has indicated that factors related to virulence in *S. aureus* are also encoded in mobile genetic elements (MGEs) like plasmids, insertion sequences (ISs), and prophages [16, 17]. A well‐known example is β‐hemolysin‐converting (or NM3) prophages (hereafter φSa3int) carrying various human immune‐evasion cluster (IEC) genes (*sak, chp, scn*, and *sea/sep)* that protect bacteria from neutrophil‐dependent phagocytosis [18, 19].

Earlier, we reported that *S. aureus* isolated from CRSwNP patients often harbored φSa3int prophages that could disrupt the production of β‐hemolysin [20]. β‐hemolysin is a sphingomyelinase hemolysin that significantly contributes to *S. aureus* pathogenesis [21], reduces the ciliary activity of nasal epithelial cells and induces sinusitis [22]. However, the pathogenic role of β‐hemolysin toxin in humans is argued due to negative conversion by the φSa3int prophage, which is present in most *S. aureus* colonizing humans [23]. Widespread distribution of φSa3int prophages among *S. aureus* isolated from the nasal cavity of humans suggests that the prophage induction and reintegration of the released phages drive dissemination of virulence genes, particularly IEC, contributing to the genetic diversification and functional adaptations of *S. aureus* [24]. However, the presence of φSa3int prophage DNA does not necessarily imply the functionality of the VFs it carries and, as such, it is important to understand the expression of prophage‐associated VFs (PVFs) and how it may play a broader role in overall bacterial gene expression and chronic infection in human sinuses. Prophage‐mediated immune evasion in *S. aureus* (particularly via φSa2int and φSa3int prophage integration) is well studied, but it is unknown how prophage domestication might affect expression of core‐bacterial genes and the expression of already domesticated residing prophages. As such, we hypothesized that *S. aureus* strain might establish itself in sinuses of chronically infected CRS patient by acquisition of highly mobile PVFs, particularly prophages encoding human IEC genes, which equips them with important virulence features.

This study aimed to investigate if φSa3int prophage transduction and encoded genes impact the phenotype and virulence of *S. aureus* isolated from CRS patients by using two genetically similar *S. aureus* isolates recovered from a severe CRS patient at different time points. To do this, we studied the effect of φSa3int prophage integration on the growth kinetics, genomics, proteomics, and capacity of macrophages to phagocytose the transformed lysogens.

## 2 Results

### 2.1 Genomic Features of SA222, SA333, Lysogens (SA‐L1 and SA‐L2), and the Prophages

**FIGURE 1:**
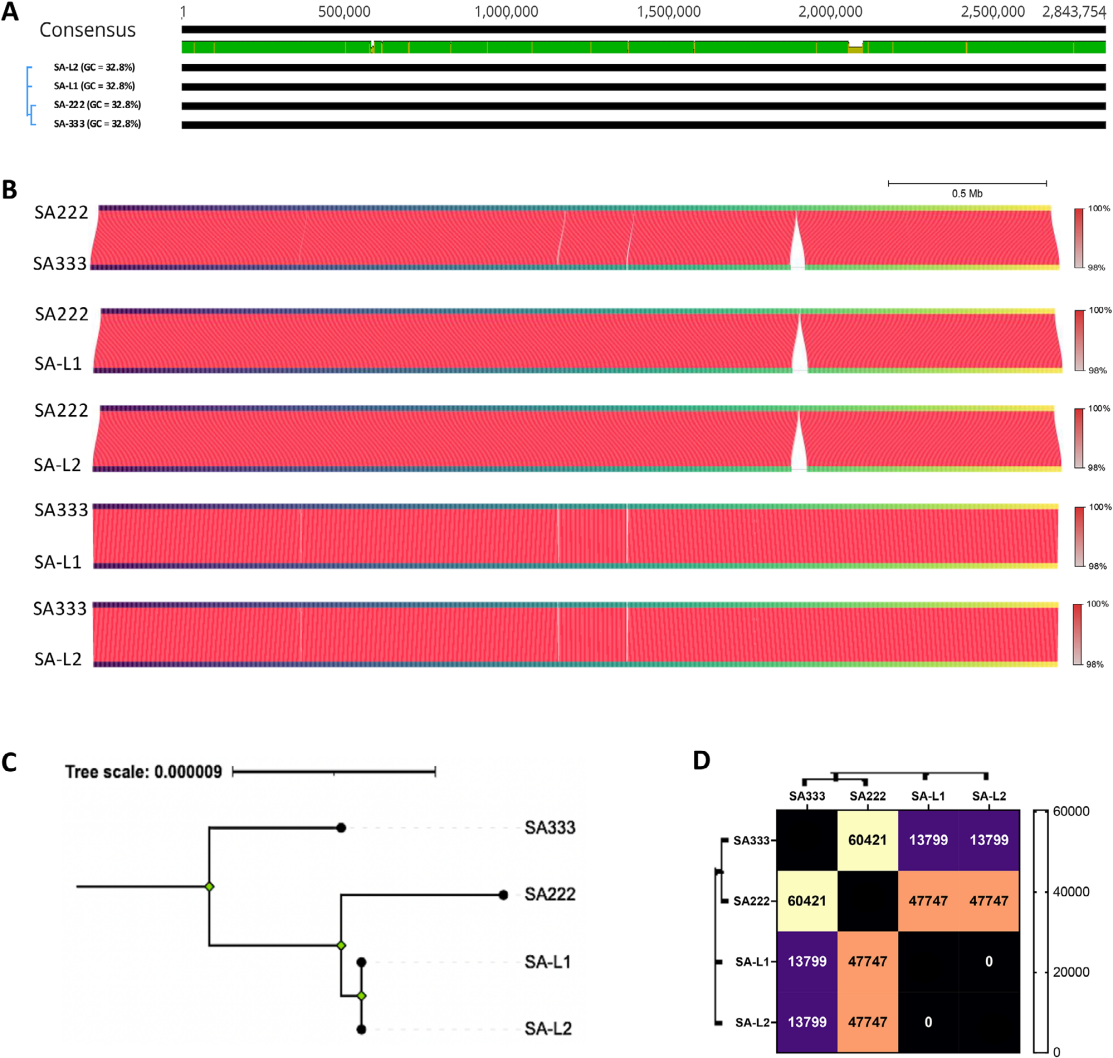
Genome alignments of clinical isolates and the lysogens. (A) Genome alignments of SA222, SA333, and laboratory generated double lysogens (two isolates; SA‐L1 and SA‐L2) show almost identical genome maps except for the prophage insert around 2.1 Mbp. (B) The average nucleotide identity (ANI) score between the clinical isolates and the lysogens further confirms that the prophage inserts on SA333 are the only significant genome change that has happened over time. The red color gradient indicates the ANI percentage. (C) A rooted phylogenetic analysis between clinical strains and laboratory generated double lysogens reveals that as expected the double lysogens are genetically closer to the recipient strain SA222. (D) Distance matrix indicating the number of bases that are non‐identical.

Sequencing and genomic analysis revealed that there were minimal genetic variations between SA222 and SA333, which were isolated from the same CRS patient 567 days apart. The strains had the same 32.8% GC content, clonal complex (CC; CC22) and sequence type (ST; ST22) (Figure 1A and Table 1). The average nucleotide identity (ANI) between SA222 and SA333 was 99.99%, and the average aligned length between the two genomes was 2,390,601 bp (Figure 1B). The alignment identified an additional φSa3int‐group prophage (hereafter φSa3int prophage) in SA333. Similarly, the ANI between SA222 (the recipient strain) and lysogenized SA222 that was laboratory‐generated by lysogenization with φSa3int prophage (SA‐L1, SA‐L2) was ∼100%, with an average aligned length of 2,746,692 bp and the ANI between donor SA333 and lysogens (SA‐L1, SA‐L2) was 99.99%, with an average aligned length of 2,385,728 bp (Figure 1B). The key difference between the clinical isolates was that while SA222 harbored one intact prophage (φSa2int, 52,500 bp), SA333 had two intact prophages (φSa2int; 50,792 bp and φSa3int; 43,795 bp) (Table 1). Upon gene annotation, we could see that although the φSa2int prophage in SA333 lost some DNA compared with SA222, SA333 had gained two transposases (length = 1236 and 768 bp) (Figure S1A,D). The identified φSa2int prophage in SA222 and SA333 was most similar to Staphylococcus phage phi2958PVL (NC_011344, 47,342 bp), while the additional prophage in SA333 was most similar to Staphylococcus phage IME1361_01 (NC_048657, 43,516 bp) (Table 1). There were only 60,421 bp of non‐identical nucleotide bases between SA222 and SA333, including the 43,795 bp φSa3int prophage (Figure 1D), confirming that SA222 and SA333 were the same strain, but had gained the flexible prophage (φSa3int) at some point in time. Phylogenetic analysis and non‐identical nucleotide differences between clinical isolates and the lysogens implied, as expected, that the laboratory‐generated double lysogens were genomically closer to SA333 than SA222 (Figure 1C,D). Surprisingly, the non‐identical nucleotide bases between the recipient SA222 and SA‐L1/L2 were 47,747 bp, which was 3952 bp larger than the exact prophage identified suggesting some auxiliary cargo DNA and rearrangements in the lysogens during prophage integration (Figure 1D).

**TABLE 1:** Details of the *Staphylococcus aureus* clinical isolates used in this study.

Bacterial strain	Collection date	Genome size (bp)	Genotypic and phenotypic characters	No of prophage^a^	Prophage type^b^ (size)/proteins	Closest prophage (NCBI reference)	Virulence genes^c^
SA222	08/01/2014	2,788,579	CC22/ST22, MRSA, high‐biofilm, hlb (+), nonmucoid	1	φSa2int (52.5 kbp)/68	Staphylococcus phage phi2958PVL (NC_011344)	*luk* SF‐PV
SA333	29/07/2015	2,837,533	CC22/ST22, MRSA, hyper‐biofilm, hlb (−), mucoid	2	φSa2int (50.8 kbp)/66	Staphylococcus phage phi2958PVL (NC_011344)	*luk* SF‐PV
					φSa3int (43.8 kbp)/69	Staphylococcus phage IME1361_01 (NC_048657)	*sak, chp, scn*
SA‐L1, SA‐L2	Laboratory generated (this study)	2,832,387	CC22/ST22, MRSA, high‐biofilm, hlb (−), nonmucoid	2	φSa2int (52.5 kbp)/68	Staphylococcus phage phi2958PVL (NC_011344)	*luk* SF‐PV
					φSa3int (43.8 kbp)/69	Staphylococcus phage IME1361_01 (NC_048657)	*sak, chp, scn*

Abbreviations: hlb (−), beta‐hemolysin absent; hlb (+), beta‐hemolysin present; MRSA, methicillin‐resistant *S. aureus*; *luk* FS‐PV, leukocidin (Panton‐Valentine FS); *sak*, staphylokinase; *chp*, chemotaxis inhibitory protein; *scn*, Staphylococcal complement inhibitor. SA‐L1 and SA‐L2 (SA222+φSa3int) are independent lysogens created by infecting SA222 with prophage induced from SA333.

^a^ Only intact prophages considered for the analysis; however, a 10,194 bp incomplete prophage encoding 17 proteins was also present in SA222 and SA333.

^b,c^ Based on *Staphylococcus aureus* integrase typing (Goerke et al., 2009).

### 2.2 Phages Induced from Clinical Isolates and the Lysogen Displayed a Multiple Host‐Range

**FIGURE 2:**
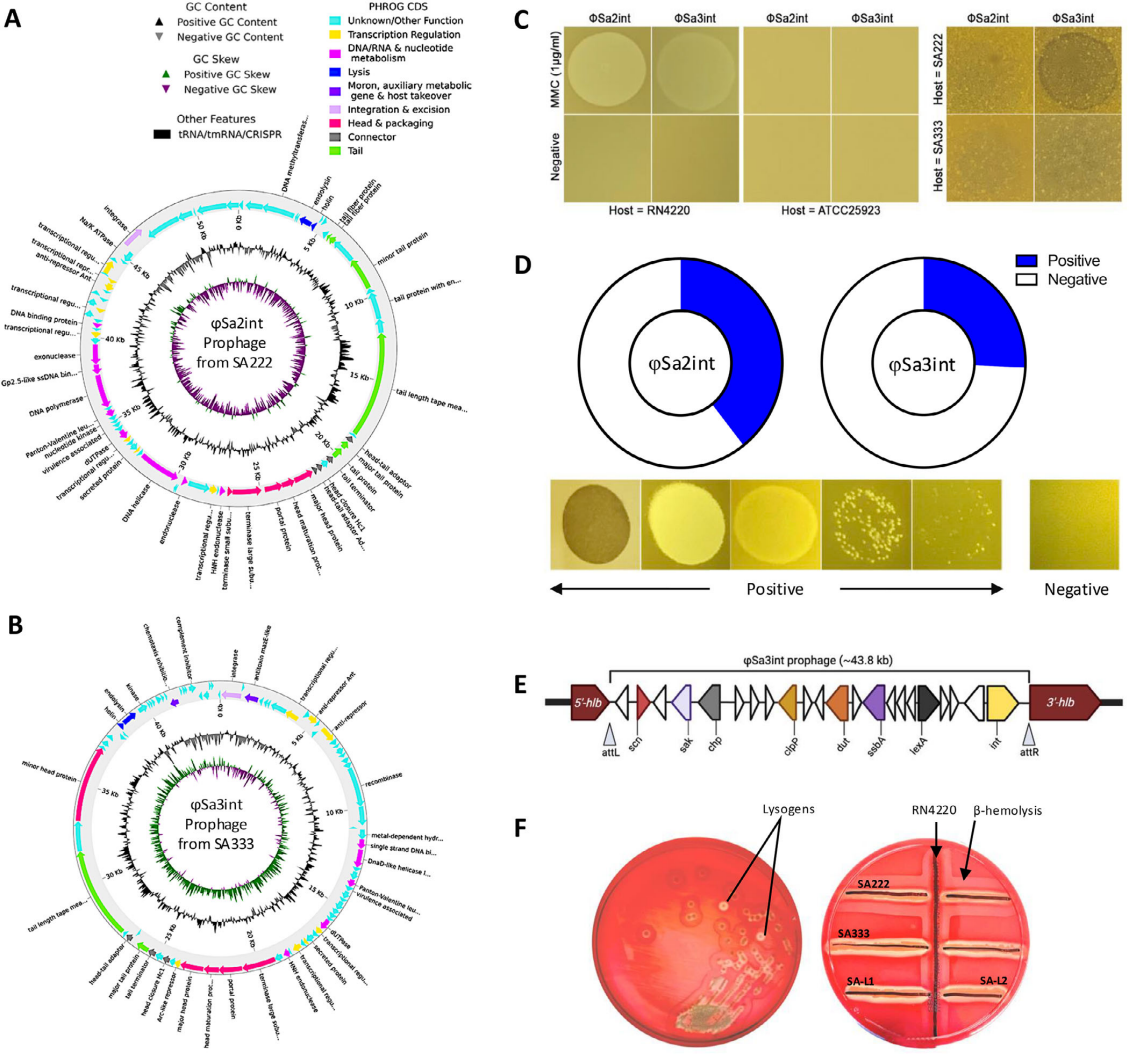
Annotation of identified prophages in SA222 and SA333 and their inducibility. (A) The φSa2int prophage is present in both clinical strains SA222 and SA333. The 52.5 kbp φSa2int prophage region primarily consists of hypothetical genes of bacterial as well as phage origin whose functions are as yet unknown. (B) The φSa3int prophage was present in clinical isolate SA333 in addition to φSa2int. The ∼43.8 kbp φSa3int prophage encoded major human immune evasion genes (*sak, scn, chp*). These genes are clustered together near the integrase (*xerC*). (C) Productive induction of prophage was observed from both clinical isolates (SA222 and SA333) using mitomycin (1.0 µg/mL). The induced phages showed clear lysis on indicator host RN4220 but did not show lysis on *S. aureus* ATCC25923. Further, the induced phage from SA333 (φSa3int) was able to lyse SA222, but the induced phage from SA222 (φSa2int) was unable to infect SA333 or SA222, possibly because of superinfection immunity. (D) The multiple host‐range of induced phages from SA222 (φSa2int) and SA333 (φSa3int) indicated that the phages can infect and kill multiple clinical isolates. The φSa2int prophage from SA222 was able to infect almost 40% (26 out of 66) clinical strains while φSa3int induced phage from SA333 could infect almost 26% (17 out of 66) clinical isolates. The lower figure panels indicate the spots that were considered positive and negative. (E) Schematic chromosomal location of transduced φSa3int prophage and identified genes in SA‐L1. The ∼43.8 kbp insert was integrated within the β‐hemolysis (*hlb)* gene thereby truncating it. (F) Assessment of β‐hemolytic activity on sheep blood agar. The modified lysogens (SA‐L1 and SA‐L2) lost their β‐hemolytic property.

The genome of φSa2int prophage mostly encoded phage‐related genes and hypothetical genes (Figure 2A). Similarly, the φSa3int prophage encoded a complete set of IEC genes (*sak, chp, scn*) and phage‐related genes (Figure 2B). Neither of the prophages encoded any ARGs. However, both prophages had a *clpP* gene encoding Clp protease involved in lysogenic‐lytic switching [25], indicating that both prophages were capable of productive induction (Figure 2A,B).

Productive prophage induction was observed in both isolates, as lysis zones (plaques) were observed on an indicator strain RN4220 in the spot assay. A clear lysis zone indicated a successful lysogenic‐to‐lytic switching of the prophages, release of productive phage particles, and a re‐infectivity of φSa2int and φSa2int and/or φSa3int (pro)phages induced from SA222 and SA333, respectively (Figure 2C). While induced prophage from SA333 (φSa2int and/or φSa3int) was able to infect and partially lyse SA222 (turbid lysis spots, lacks φSa3int prophage), induced prophage from SA222 (φSa2int) could not infect or lyse SA333 (which already has both φSa2int and φSa3int prophages), likely because of superinfection exclusion (Figure 2C). Similarly, induced prophage from lysogens (SA‐L1 and SA‐L2) could infect the parent strain SA222 and RN4220 but not SA333, indicating productive induction of transduced φSa3int prophage.

Further, released phages from both clinical isolates (SA222 and SA333) produced partial to complete lysis spot on multiple clinical isolates in spotting assay, indicating multiple host‐range of the induced prophages and their ability to transduce PVFs to multiple clinical isolates. Representative negative and positive lysis spots are shown in (Figure 2D). Prophages induced from SA222 (φSa2int) could produce lysis spots on 39.4% (26 out of 66) of the tested clinical isolates, while productive prophages from SA333 (either φSa2int or φSa3int) could produce lysis spots on 25.8% (17 out of 66) of clinical isolates (Figure S3). Phages released from both clinical isolates did not lyse *S. aureus* ATCC25923 and other clinical isolates that contained both residing φSa2int and φSa3int prophages (8 out of 66 isolates) (Figures 2C and S3) in intact or incomplete form.

### 2.3 Integration of φSa3int Prophage Inhibited the Production of β‐Hemolysin

Whole genome sequencing of the lysogens (SA‐L1 and SA‐L2) confirmed that a ∼43.8 kbp φSa3int prophage DNA induced from SA333 was integrated into the chromosome of SA222 within the *hlb* gene (start = 2,041,825 bp, end = 2,088,955 bp) (Figure S1), generating a double‐lysogen with negatively converted *hlb* gene and incorporation of all three IEC genes (*sak, scn*, and *chp*) (Figure 2E). The *hlb* gene was truncated near the 5’ end resulting in two incomplete *hlb* gene remnants; 201 bp (66 aa; 7,321.09 amu) and 825 bp (274 aa; 31,257.36 amu) (Figure S2). As such, the lysogens lacked β‐hemolytic activity in sheep blood agar (SBA) (Figure 2F). Genomic analysis also revealed that the φSa3int prophage in this study lacked *sea/sep* genes, which are commonly found PVFs in Sa3int‐group prophages (Figure 2B).

### 2.4 Domestication of a φSa3int Prophage did not Impact Growth Kinetics, Biofilm Biomass, Metabolic Activity, and Adhesion to Human Nasal Epithelial Cells

**FIGURE 3:**
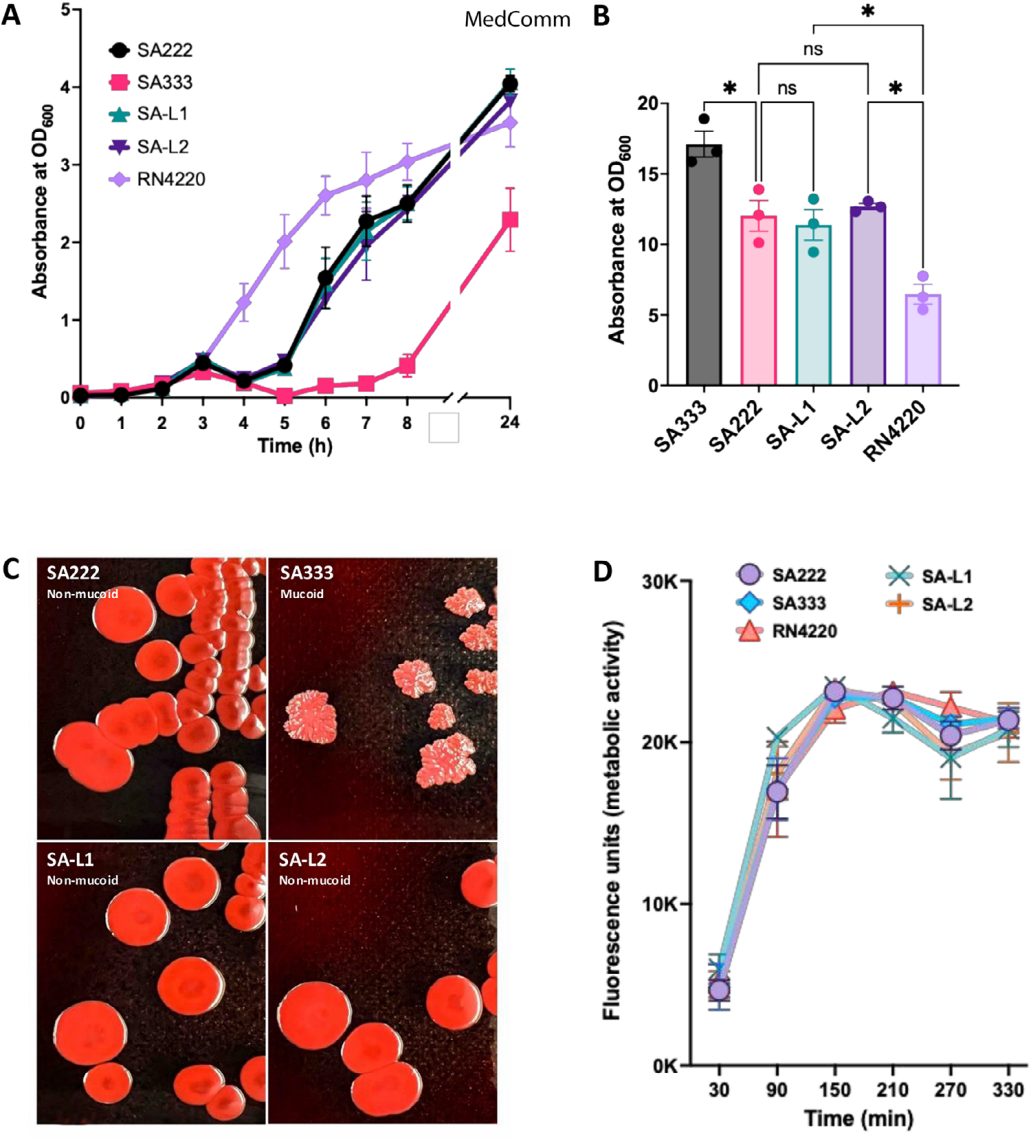
Comparisons of in vitro characteristics between SA222, SA333, and laboratory generated double lysogens (SA‐L1 and SA‐L2). (A) Bacterial growth curve in tryptic soy broth did not show any significant change in growth kinetics between recipient SA222 and double lysogens (SA‐L1 and SA‐L2), indicating domestication of an additional ∼43.8 kbp φSa3int prophage did not impact the growth of the recipient strain SA222. (B) Biofilm estimation by crystal violet assay also indicated that the domestication of ∼43.8 kbp φSa3int prophage did not impact biofilm formation. (C) Overnight culture of *S. aureus* strains in Congo red for qualitative estimation of mucoid phenotype further indicated that the φSa3int prophage and the genes it carried had no impact on phenotypic differentiation between mucoid and non‐mucoid phenotype. (D) Study of biofilm metabolic activity by alamarBlue cell viability assay indicated that there was no significant change in metabolic activity between recipient isolate (SA222) and double lysogens (SA‐L1 and SA‐L2).

The domestication of the additional ∼43.8 kb φSa3int prophage DNA did not alter lysogen's growth‐kinetics compared with the recipient SA222 (Figure 3A). Also, there was no significant change in biofilm biomass between the lysogens and SA222 (Figure 3B). The colonies of SA222 and SA‐L1/SA‐L2 on Congo red agar (CRA) were smooth indicating non‐mucoidy. In contrast, the colonies of donor SA333 on CRA was wrinkled, suggesting mucoid production (Figure 3C). Further, the metabolic activity of the biofilm was also similar between lysogens and recipient SA222 (Figure 3D). However, the biofilm biomass and biofilm metabolic activity of SA333 (donor) were significantly higher than the laboratory‐generated double lysogens (SA‐L1/SA‐L2), despite having non‐aligned nucleotide differences of 13,797 bp only (Figure 1B,D). Further, there was no significant difference in adhesion of recipient SA222 and the double lysogens in primary human nasal epithelial cells (HNEpCs). The relative adhesion of the lysogen to the HNEpCs compared with the recipient host SA222 was 94%, implying a slight reduction in the adhesion of *S. aureus* after infection with φSa3int prophage but did not reach statistical significance (Figure S4).

### 2.5 Integration of φSa3int Prophage Added Human Immune Evasion Factors to *S. aureus*

**FIGURE 4:**
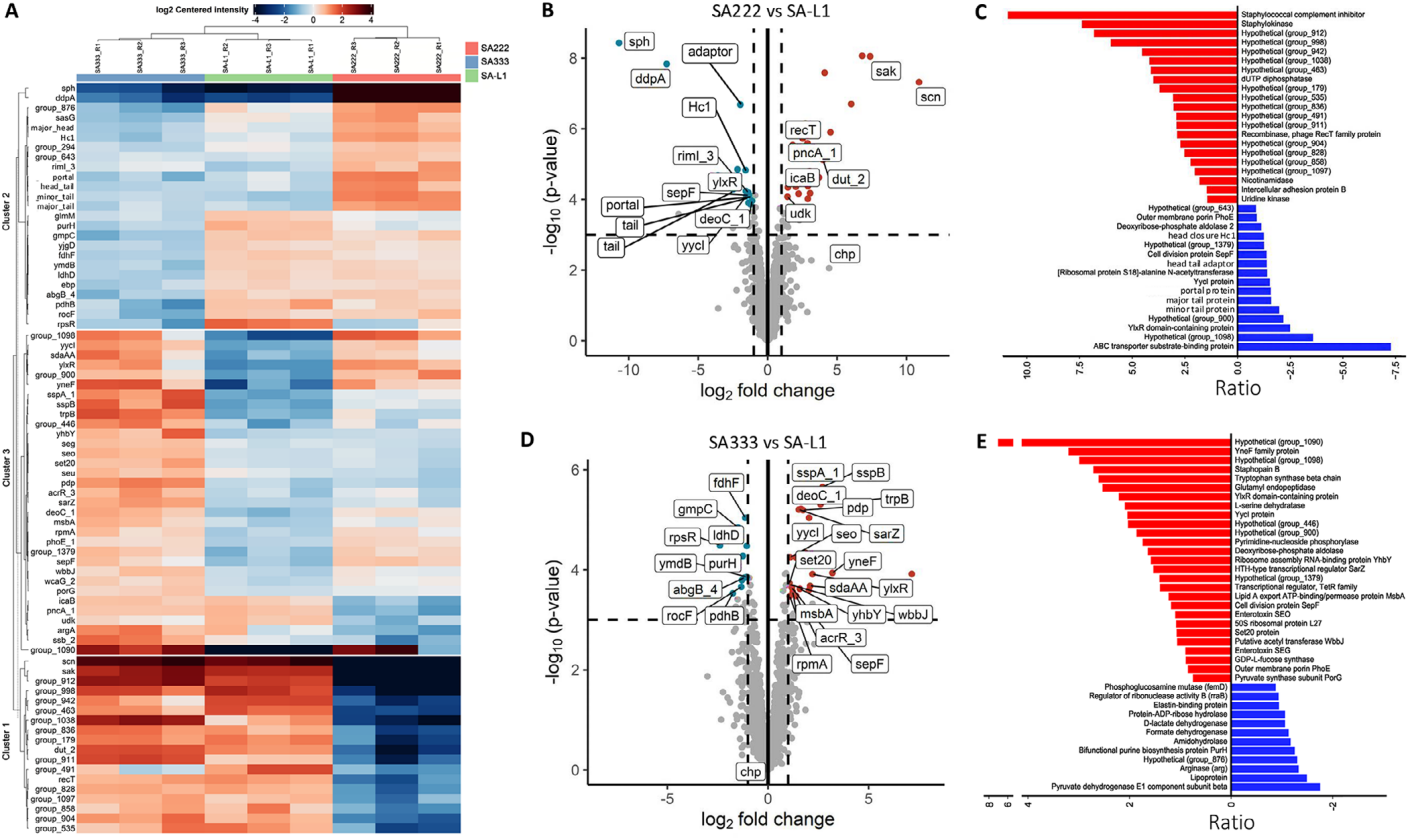
Proteomics of the secretome from SA222, SA333 and SA‐L1. (A) The heatmap showing the differential expression between donor SA333, recipient SA222 and laboratory‐generated double lysogen (SA‐L1). The proteins in cluster 1 (including Sak, Scn, RecT, Dut) were significantly upregulated in SA‐L1 compared with recipient isolate SA222. The expression of genes in this group was similar between the lysogen and the donor isolate SA333. (B) Volcano plot showing the differential expression of proteins between recipient host SA222 and lysogen (SA‐L1). (C) A bar plot showing the relative ratio of significantly up‐ and down‐regulated proteins between SA222 and SA‐L1. The virulence factors responsible for human immune evasion (Sak and SCIN) are significantly upregulated along with the intercellular adhesion protein B (IcaB). (D) Volcano plot showing the differential expression of proteins between donor host SA333 and lysogen (SA‐L1). (E) A bar plot showing the relative ratio of significantly up‐ and downregulated proteins between SA333 and SA‐L1. There was no significant change in virulence factors encoded by prophage φSa3int, indicating the difference in phenotypes is not associated with prophage‐associated genes or functions. *p* < 0.05 was considered significant in all the above analyses.

The proteomics of the secretome collected from SA222, SA333 and one of the laboratory‐generated double lysogens (SA‐L1) indicated that acquisition of the φSa3int prophage armed *S. aureus* bacteria with multiple PVFs including human IEC factors. These include staphylokinase, Staphylococcal complement inhibitor (SCIN), a chemotaxis inhibitory protein of *S. aureus* (CHIPS), and recombinase protein (RecT) that are secreted as exoproteins (Figure 4A, cluster 1). Altogether, 37 exoproteins were differentially expressed in SA‐L1 compared with its recipient strain SA222. Among them, 21 (55.3%) were upregulated including staphylokinase (Sak), SCIN (or Scn), and intercellular adhesion protein B (IcaB). In contrast, 16 proteins (44.7%) were downregulated including β‐hemolysin (Hlb/Sph) and outer membrane porin (PhoE) (Figure 4B,C). Among 16 of the downregulated proteins, 5 (31%) (mostly head and tail structural proteins) were encoded by the resident φSa2int prophage SA222. This clearly indicated that, during bacterial adaptation via successful domestication of a new prophage, the incoming prophage (φSa3int) not only diversified the bacteria by adding prophage‐encoded genes but also suppressed expression of the prophage genes (φSa2int) already present in the bacteria. Functions of 17 proteins (∼46%) were unknown (hypothetical). Similarly, despite having few genetic variations between donor host SA333 and the lysogen SA‐L1 (Figure 1B,D), 40 proteins were differentially expressed in SA‐L1 (Figure 4A, clusters 2 and cluster 3). Among them, 27 (67.5%) including Staphylococcal enterotoxin O (SEO) and SEG (responsible for inducing inflammatory response and pathogenesis, respectively) were upregulated in lysogen SA‐L1 compared with SA333 indicating enhanced inflammatory response by newly lysogenized strain, which may subside (downregulate) later when the lysogen establishes itself (SA333). Thirteen proteins (32.5%), including elastin binding protein (Ebp) were downregulated in laboratory‐generated lysogen SA‐L1 compared with patient‐derived SA333 (Figure 4D,E). As predicted, most of these differentially expressed proteins (DEPs) were non‐prophage encoded (bacterial origin) and hypothetical (unknown function) proteins constituted only 15.0% (6 out of 40) of proteins. This was expected as both strains (SA333 and SA‐L1) carried identical MGEs (Figure 1B).

**FIGURE 5:**
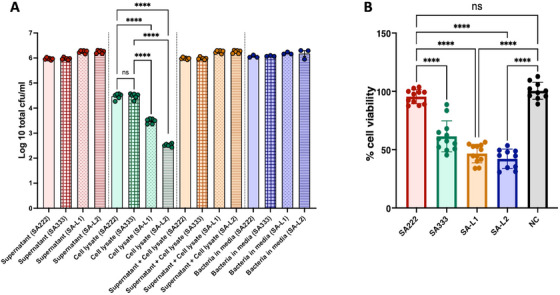
Phagocytosis and cell viability assay. (A) The number of bacterial cells recovered from lysogens SA‐L1 and SA‐L2 infected THP‐1 cell lysate was significantly lower compared with the THP‐1 cells infected with recipient SA222 indicating heightened immune evasion by lysogens. (B) The cell viability of THP‐1 cells was significantly reduced by φSa3int prophage incorporated double lysogens (SA‐L1 and SA‐L2) compared with SA222 (recipient) indicating higher cytotoxicity of φSa3int prophage harboring double lysogens. NC = negative control (THP‐1 cells without bacteria). *p* < 0.05 was considered significant in all the above analyses.

### 2.6 Domestication of φSa3int Prophage Reduces the Phagocytosis of *S. aureus* by Macrophages

The ability of macrophages to phagocytose SA222, SA333, SA‐L1, and SA‐L2 was estimated upon incubation of the bacteria with THP‐1 cells for 1 h. There was no significant difference in colony forming units (CFU) counts between bacteria incubated in media and total bacteria harvested from supernatants and cell lysates for any of the isolates tested (*p* > 0.05) (Table S1) indicating that macrophages did not affect the viability of any of the isolates tested. There was a significant reduction in average CFUs of SA‐L1 (log_10_ reduction = 0.99 and 1.95) and SA‐L2 (log_10_ reduction = 1.95 and 0.96) in cell lysates compared with SA222 and SA333 in cell lysates, respectively. In contrast, there was a significant increase in CFUs in supernatants of SA‐L1 and SA‐L2 compared with C222 and C333 (Figure 5A). These results indicate a reduction in the ability of THP‐1 cells to phagocytose the lysogens SA‐L1 and SA‐L2 compared with recipient SA222 and donor SA333. Results of CFU counts are detailed in Table S1. Further, cell viability of THP‐1 cells was evaluated by quantification of lactate dehydrogenase (LDH) release after exposure to SA222, SA333, SA‐L1, and SA‐L2 at multiplicity of infection (MOI) 50 for 1 h. No significant difference in LDH levels was seen in SA222 infected cells compared with the negative control; however, cell viability was reduced by up to 42% when THP‐1 cells were incubated with SA333, SA‐L1, and SA‐L2 (Figure 5B). These results indicated that insertion of prophage into SA222 increased the cytotoxicity of the lysogens SA‐L1 and SA‐L2 to THP‐1 cells.

## 3 Discussion

The productive induction of prophages from a lysogen leads to the release of active phage particles that can lysogenize susceptible bacterial strains [26]. It is established that various drugs, antibiotics, and even dietary compounds enhance prophage induction and promote lysogenic conversion [6, 8, 27]. Prophages can also be induced spontaneously in response to nutrient availability and/or other environmental conditions, including niche variation. As over 90% of human clinical isolates carry Sa3int‐group (*Staphylococcus aureus* type‐3 integrase) prophages predominantly integrated into the *hlb* gene [18], we aimed to elucidate the contribution of φSa3int prophage domestication in the *S. aureus* secretome. To achieve this, we introduced a φSa3int prophage (also called β‐hemolysis‐converting prophage) induced from a patient‐derived double‐lysogen (SA333), into a genetically similar single (φSa2int)‐lysogen (SA222), isolated from the same patient almost 2 years earlier, to create a laboratory‐generated double‐lysogen that harbors both φSa2int and φSa3int prophages. We then studied its phenotypic characteristics, genomics as well as proteomics. We chose these clinical isolates for our experiments because, our preliminary study suggested that although there were significant phenotypic variations between SA222 (high‐biofilm, hlb (+)) and SA333 (hyper‐biofilm, hlb (−)), the two CIs were genetically similar except for the gain of φSa3int prophage by SA333 and also were collected from the same patient who had severe CRS. As phage infection is highly specific, it is important to select genetically similar strains for uncovering the role of prophages.

Lysogenic conversion in *S. aureus* arms bacteria with many survival fitness traits like VFs, toxins, and biofilm upregulation and is common in clinical strains [28, 29, 30]. Prophage‐mediated enhancement of biofilm has been observed in various bacteria like *Enterococcus faecalis* [31], Streptococcus *pneumoniae* [9], *Escherichia coli* [32], and *S. aureus* [28]. Further, multiple studies have described prophage‐mediated phenotypic alteration, host adaptation, and pathogenesis [33, 34, 35]. Our findings support the notion that lysogenic conversion in clinical isolates is common [24, 36], as almost 40% of the CIs tested could be infected with φSa2int and/or φSa3int released from an unrelated clinical isolate, meaning they could gain PVFs and pathogenicity via prophage domestication. Further, the variations in the strength of lysis spots in multiple host‐range assays indicated that the induced phage particles (temperate) had varying degrees of susceptibility among CIs, suggesting variable lysogenic conversion of the host. The susceptibility to φSa2int and/or φSa3int was absent in all isolates that carried both φSa2int and φSa3int prophages, likely due to superinfection exclusion, but susceptibility appeared not related to CC or STs. However, as most of the isolates in this study had multiple prophage‐type integrated [36], future research with bacterial isolate groups with only specific prophage type is required to ascertain the specificity, rate of lysogenic conversion, and expression of PVFs.

Several studies on *S. aureus* clinical isolates have proposed hemolysin (Hlb) as an important nonpore‐forming toxin promoting colonization and impacting ciliary clearance of bacteria in an animal model [37, 38]. A similar function of Hlb has thus been speculated in humans. However, most human‐associated *S. aureus* lack β‐hemolysin because of the integration of the Sa3int (or NM3) prophage in the *hlb* gene [18, 20]. A similar truncation of the *hlb* gene in this study is seen after integrating φSa3int prophage DNA, completely disrupting the production of the Hlb protein in the secretome. This seems counterintuitive as Hlb is a strong sphingomyelinase that promotes sphingomyelin degradation and stimulates biofilm formation in the presence of extracellular DNA (eDNA) [39, 40]. Thus, we speculate that nasal colonizers might trade‐off the Hlb function by gaining immune evasion factors that are encoded by the φSa3int prophage and could protect bacteria from phagocytosis [41, 42]. Our findings support this as φSa3int prophage integration significantly reduced phagocytosis by macrophages in the in vitro setting. Re‐expression of Hlb has been noted in *S. aureus* isolated from cystic fibrosis patients upon antibiotic (ciprofloxacin or trimethoprim) treatment and increased frequency of genomic alterations have been associated with prophage mobilization [43]. Other studies have shown the conditional excision of φSa3int prophage in a sub‐set of the population in in vivo conditions and *S. aureus* thriving as a heterogeneous population that aggravates the infection [44]. As the laboratory generated φSa3int prophage in our study could be reinduced, we support the notion that φSa3int prophage integration and excision are conditional and largely depend on external factors. However, more in vivo study is required to confirm the hypothesis and understand the conditional switching of *S. aureus* from Hlb positive to negative or vice versa.

*S. aureus* biofilm and its adhesion to various surfaces are important features that aid bacteria in colonizing and establishing themselves in various niches. Fernandez et al. [28] suggested the role of *S. aureus* prophages (φ11 and φ80α) in biofilm development and adhesion via activation of the SigB regulon. Other studies have suggested that the eDNA released during SPI acts as a quorum sensor leading to an enhanced biofilm formation [9]. However, we did not observe significant enhancement of biofilm production or adhesion to HNEpCs when comparing SA222 and SA‐L1/L2, although biofilm‐associated icaB was upregulated in the lysogen SA‐L1 compared with SA222. The contradictory findings may be due to the different prophage type used for lysogenization because Fernandez et al. [28] used φ11 (Sa5int‐group) and φ80 (Sa6int‐group) to lysogenize the laboratory strain RN4220, while we used a Sa3int‐group prophage to lysogenize another clinical isolate (SA222), which already was high‐biofilm forming strain and as such the difference in biofilm formation was non statistically significant. Of note, when comparing the secretome of SA333 with that of LA‐L1/L2, 15% of DEPs were of unknown function. Unravelling their function might help explain the difference in biofilm forming capacity and switch to the mucoid phenotype. Although the result was unexpected, the observation ruled out a major contribution of φSa3int prophage including all the hypothetical genes it carries in biofilm development, adhesion, and phenotypic difference between *S. aureus* SA222 and SA333. Since patient‐derived SA333 and laboratory‐generated double‐lysogens were genetically almost identical (99.51 % similar) except for a few single nucleotide polymorphisms (SNPs) and ISs (data not shown) but had different biofilm, adhesion, and mucoid phenotypes, the result has helped us narrow down on other possible genes responsible for hyper‐biofilm formation and mucoid phenotype in *S. aureus*. Further mutagenesis studies are required to evaluate the molecular basis of hyper‐biofilm production and mucoid phenotype switching of the SA333 isolate.

Further, our results suggest that although integration of a Sa3int‐group prophage seems expensive in terms of replication energy cost owing to its ∼43.8 kb size and disruption of the β‐hemolysin expression, the prophage equips the host bacteria with a multitude of accessory VFs like sak, scn, chp, icaB. The high incidence of human‐specific anti‐innate immunity factors in *S. aureus* isolated from humans is well known [42]. It is also established that φSa3int prophages carry and disseminate IEC genes (*sak, chp, scn, sea/entA*), either partial or a complete set [42]. In vitro secretome profiling of the SA222, SA333, and lysogen (SA‐L1) revealed major changes in virulence, particularly human immune evasion modulation of *S. aureus* associated with the domestication of the prophages. Our results confirm and extend the existing knowledge that φSa3int‐group prophages carry IEC genes in a cluster that are significantly upregulated and secreted as exoproteins by the bacteria, which then collectively assist in persistent colonization of the bacteria by helping bacteria escape the human innate immune system. Staphylokinase protein (Sak) encoded by *sak* gene is a potent plasminogen activator that converts plasminogen into plasmin. Sak‐mediated plasmin activity increases the local invasiveness of *S. aureus* leading to skin disruption and reduced clearance of bacteria by the host [45]. CHIPS encoded by *chp* gene counters the first line of host defense, specifically inhibiting the response of human neutrophils and monocytes to complement anaphylatoxin C5a and formylated peptides. It directly binds to the C5a and formylated peptide receptors, preventing phagocytosis of the bacterium [46]. Similarly, SCIN protein encoded by *scn* gene also counters the first line of host defense. It efficiently inhibits opsonization, phagocytosis, and the killing of *S. aureus* by human neutrophils [47]. All of these genes were significantly upregulated in the lysogen secretome along with downregulation of hemolysin, indicating a quadruple conversion of isolate SA222 by φSa3int prophage induced from SA333. These results also hint that the patient isolate SA222 gained a φSa3int prophage and established itself in the nasal niche of the patient, as multiple CIs isolated after this time point were almost identical with both φSa2int and φSa3int stably integrated into the host's genome with a complete set of IEC genes (data not shown). Further, multiple structural proteins encoded in φSa2int prophage (major head protein, head‐tail adaptor, head closure Hc1, major and minor tail proteins) were significantly downregulated in laboratory‐generated double‐lysogen SA‐L1 compared with recipient SA222 indicating suppression of productive induction of resident prophage (φSa2int) after the isolate (SA222) gaining new (φSa3int) prophage. This suppression might help prevent conflicts between the different resident prophages allowing their stable coexistence within the host bacterium over time. Whilst mechanisms of defense against exogenous phage attack by resident prophages have been investigated in Gram‐negative pathogens [48], further experiments are required to investigate the molecular interactions of prophage acquisition and how they affect resident prophages in *S. aureus*.

Together, our findings indicate that despite an increase in the genome size of the bacterium by almost 43.8 kb of Sa3int‐group prophage, the prophage integration alone does not significantly alter bacterial growth kinetics, biofilm formation, and adhesion to HNEpCs in *S. aureus*. However, the proteomics analysis of the secretome clearly indicates that lysogenization by Sa3int‐group prophage arms the bacteria with additional key virulence features likely helping bacteria evade the human immune system by avoiding phagocytosis and later adapt to the niche. Further, as induced phages could infect other clinical isolates but not their own parental host, we can assume that prophage domestication not only increases the virulence of the lysogenized host but also can landscape the microbiome, which may lead to bacterial dysbiosis and ultimately pathological conditions. Temperate phages (which are often released by productive prophage induction) have been observed in various environments like human gut, chronic wounds, and cystic fibrosis patients aggravating the disease. Recent advances in genomic sequencing have revealed that most *S. aureus* clinical isolates adapted to human's harbor diverse prophages in their genome, highlighting the importance of testing prophage‐encoded toxigenic traits in chronic diseases like CRS. Further, understanding prophage‐driven coevolution and persistence is clinically important to optimize therapeutic strategies for chronic infections. In addition to CRS, the role of temperate phage or prophage is being more evident in various chronic diseases like chronic wounds, inflammatory bowel disease, and cystic fibrosis, which indicates prophages might be one of the important hidden MGEs shaping bacterial virulence and pathogenicity.

In summary, we conclude that lysogenic conversion of *S. aureus* by φSa3int (also known as NM3) prophage alters the bacterium's virulence by upregulating the human immune evasion factors like Sak, Scn, and Chp and downregulating β‐hemolysin, a sphingomyelinase. Our research further confirmed that the growth, biofilm, and adhesion of *S. aureus* are not associated with Sa3int‐group prophage domestication but significantly reduces the uptake by macrophages, which may lead to chronic infection and possibly plays role in severity of CRS and other chronic infections. These findings demonstrate the need to consider MGEs like prophages while developing a treatment strategy in chronic diseases like CRS, as strains with or without prophages have different virulence properties and risks of chronic colonization, despite having almost identical core genomes.

### 3.1 Limitations and Future Direction

Although we could affirm the origin of human IEC genes in *S. aureus* and predict the location of φSa3int‐group prophage insertion with accuracy, we could not identify the genes responsible for increased biofilm in SA333. However, the successful introduction of φSa3int prophage into SA222 ruled out the role of prophage and prophage‐associated genes in the high‐biofilm phenotype of *S. aureus* SA333, redirecting future research to the limited number of SNPs and ISs that have been identified (unpublished thesis, data not shown) and ISs that are present on SA333 but not on the lysogens (SA‐L1) and SA222. Also, the hypothesis can be further expanded to other types of prophages that carry different sets of VFs.

## 4 Materials and Methods

### 4.1 Bacterial Strains, Cells, and Growth Conditions

All *S. aureus* CIs were retrieved from glycerol stocks and cultured at 37°C overnight on nutrient agar (NA; Oxoid Ltd, Hampshire, UK). *S. aureus* RN4220 and *S. aureus* ATCC25923 were from the German Collection of Microorganisms and Cell Cultures (DSMZ, GmbH) and American Type Culture Collection (ATCC, Manassas, USA), respectively. The isolates SA222 and SA333 (recovered from a severe CRS patient at different time points) were selected based on the prior clinical history on this patient, genomic similarity (identical core genome but an extra φSa3int prophage in SA333), and extremely high‐biofilm forming nature of both SA222 and SA333 compared with other CIs. The first isolate (SA222) was high‐biofilm forming non‐mucoid with only φSa2int prophage while the SA333 was hyper‐biofilm forming mucoid isolate with an additional φSa3int prophage that replaced SA222 and chronically infected the patient (Table 1). The HNEpCs used for the adhesion assay were collected from a non‐CRS (control) patient at the time of surgery.

### 4.2 Genomic DNA Extraction, Sequencing, and Genome Assembly

The genomic DNA (gDNA) of all *S. aureus* CIs were extracted using DNeasy Blood & Tissue Kit (Qiagen; #Cat: 69504) according to the manufacturer's guidelines. The extracted gDNA was sequenced using the short‐read Illumina NextSeq 550 platform using NextSeq 500/550 Mid‐Output kit (v2.5) (Illumina Inc, San Diego, USA) at a commercial sequencing facility SA Pathology (Adelaide, SA, Australia) and in‐house long‐read Oxford Nanopore Technology using the MinION Mk1C device (Oxford Nanopore Technologies, Oxford, UK) using MinION flowcells (R9.4.1) with Rapid Barcoding Kit (Oxford Nanopore Technologies; #Cat: SQK‐RBK 110.96) following the manufacturer's instructions and in‐house protocol [49]. Complete chromosomal *S. aureus *assemblies were created using Hybracter (v0.1.0) [50], reoriented with Dnaapler (v0.4.0) [51] and annotated with Bakta (v1.6.1) [52]. All isolates were typed to determine ST and CC according to the PubMLST database using MLST [53, 54]. A detailed method of this section is available as *Method 4.2* (Supporting Information).

### 4.3 In Silico Identification of Prophage and Prophage Annotation

Prophage regions in both *S. aureus* CIs (SA222 and SA333, isolated from the same patient at different time points) were first identified using PHASTEST and PhiSpy (v4.2.20) with default settings [55, 56, 57]. The exact genome of the φSa3int prophage between *hlb* gene was then curated with our in‐house program hlbroken (https://github.com/gbouras13/hlbroken). The identified φSa2int and φSa3int prophage sequences were annotated and visualized with Pharokka (v1.7.5) [58]. Prophage regions in all CI's used for multiple host‐range in this study was identified using PHASTEST and their detailed features are already published [36].

### 4.4 Prophage Induction and Multiple Host‐Range Assay of Induced Phages by Spotting Assay

Prophages from both CIs were induced using mitomycin C (MMC), purified and spotted on previously studied *S. aureus* CIs (*n* = 66) using the soft‐agar overlay assay as described elsewhere [59]. Briefly, MMC (final concentration = 1.0 µg/mL) (Sigma–Aldrich, USA; #Lot: SLBX4310) was added to exponentially growing bacterial cells (OD600 = 0.3) in TSB and incubated for 6 h at 37°C. The culture was then centrifuged (3220xg, 15 min at 4°C) and the supernatant was filter‐sterilized by passing through 0.2 µm syringe filter (13 mm; Acordisc^®^; Pall International, Switzerland; #Cat: 4612) to obtain pure phage lysate. 10.0 µL of purified lysate was spotted on the top agar of a double‐layer agar plates seeded with the test bacteria. The assay was performed in triplicates.

### 4.5 Lysogenization of SA222 with φSa3int Prophage and Verification of Successful Integration

Ten microliters of purified phage lysate induced from SA333 were spotted on the top agar of double‐layer agar plates seeded with recipient host (SA222). The plates were dried and incubated overnight at 37°C. The next day, a loopful of bacteria from the center of the lysis spots were streaked onto SBA (ThermoFisher, Australia; #Cat: R01202) and incubated overnight at 37°C. Colonies without beta‐hemolysis (potentially lysogens) were picked and sub‐cultured in SBA. The stability of these constructs possibly harboring both resident φSa2int and transduced φSa3int prophages was confirmed through multiple sub‐cultures in SBA and verification of loss of beta‐hemolysis. Two *S. aureus* constructs devoid of beta‐hemolysis (hereafter lysogens SA‐L1 and SA‐L2) were picked for further analysis. The integrity and re‐inducibility of integrated prophage were then confirmed by re‐induction from the constructs using MMC as described earlier and spot assayed on SA222, SA333, and RN4220. The integration of φSa3int prophage into the *hlb* gene was verified by inspecting the *hlb* gene in assembled genomes using the Integrative Genomics Viewer [60].

### 4.6 Growth Curve, Biofilm Biomass, Biofilm Metabolic Activity, and Adhesion Assay of *S. aureus*

Bacterial growth kinetics were determined by measuring the optical density of broth culture at 600 nm (OD600). Briefly, 100 µL of 1.0 McFarland standard unit (MFU in saline, prepared from overnight cultured colonies on NA plates) was added to 15.0 mL of TSB and incubated at 37°C in a shaking incubator (180 rpm). Every hour, 100 µL of culture was removed and mixed with 900 µL of sterile TSB in a cuvette. The OD600 was then measured using a SmartSpec 3000 UV/Vis spectrophotometer (Bio‐Rad Laboratories Inc, USA). The biofilm variation between the clinical isolates and lysogens was qualitatively assessed by culturing the bacteria on modified CRA (37 g/L brain heart infusion broth supplemented with 50 g/L sucrose, 0.8 g/L Congo red stain, and 1.0 % agar) according to Freeman et al [61]. Colony morphology of SA222, SA333, and SA‐L1/SA‐L2 on CRA was assessed after 48 h incubation at 37°C. Further, biofilm biomass and biofilm metabolic activity (cell viability) was performed using microtiter crystal violet assay and alamarBlue^®^ cell viability assay respectively (Life Technologies, USA) in biofilms established for 48 h in 96‐well flat‐bottomed (Costar, Corning Incorporated, USA; #Ref: 3599) and 96‐well flat and clear bottom black assay plates (Costar, Corning Incorporated; #Ref: 3603) respectively, as described earlier [62]. The adhesion of *S. aureus* clinical strains and lysogens to HNEpCs was studied following the protocol by Yang and Ji [63]. The detail method of this section is elaborated in *Method 4.6* (Supporting Information).

### 4.7 Proteomics of the Secretome

The proteomics of the secretome was analyzed using a data‐independent acquisition mass spectrometry (DIA‐MS) using Orbitrap Fusion Lumos Tribrid Mass Spectrometer with Dionex UltiMate 3000 UHPLC system (both from Thermo Fisher Scientific, USA). Briefly, 100 µL (1.0 MFU standard) *S. aureus* were added to 15.0 mL of TSB and incubated in a shaking incubator at 37°C (180 rpm). After ∼7 h, the tube was briefly vortexed and centrifuged at 4000×*g* for 10 min. The secretome was passed through a 0.2 µm syringe filter (25 mm; Acordisc^®^, Pall International, Switzerland) and concentrated using a Pierce Protein Concentrator PES (3K MWCO; #Cat: 88525; ThermoScientific, USA) to ∼2 mL. The protein concentration was determined using NanoOrange Protein Quantitation Kit (Invitrogen, USA; #Cat: N6666) and processed at the Flinders Omics Facility, Flinders University according to the established protocol. The DIA spectra were then processed using Spectronaut (v15) (Biognosis AG, Schlieren, Switzerland) with default settings and a manually curated *S. aureus* proteome database created from all genes identified in SA333 as a reference. Gene annotations were assigned based on SA333 strain using Bakta (v1.6.1) [64]. Differential protein expression analysis was performed in R (v4.2.0) using the DEP package (v1.20.0) to calculate DEPs [65]. The threshold for identifying DEPs was set at a false discovery rate of less than 0.05. The complete set‐up and protocols are elaborated in *Method 4.7* (Supporting Information).

### 4.8 Bacterial Uptake by Macrophage Assay

Bacterial uptake by macrophages was evaluated through THP‐1 cell line exposure to SA222, SA333, SA‐L1, and SA‐L2 at MOI of 50. Briefly, THP‐1 cells (ATCC) were seeded at 1.5 × 10^5^ cells in 750 µL RPMI containing 200 ng/mL phorbol 12‐myristate 13‐acetate (Sigma–Aldrich, St Louis, USA) in a 24‐well cell culture plate and incubated at 37°C for 48 h in a fully humidified incubator at 5% CO_2_. Cells were infected with SA222, SA333, SA‐L1, and SA‐L2 at MOI 50 for 1 h. Cells exposed to media only were considered negative control and bacteria incubated in media were used as bacterial infection control. Supernatants were collected and the remaining extracellular bacteria were removed using lysostaphin (10 µg/mL) (Sigma–Aldrich) for 15 min. To assess intracellular infection, cells were lysed with 0.1% triton X‐100 for 30 min. Serial dilution of supernatants and cell lysates was prepared in sterile saline and plated on TSA. CFUs were enumerated after overnight incubation at 37°C. Experiments were repeated three times.

### 4.9 Lactate Dehydrogenase Cytotoxicity Assay

The supernatants were collected after exposure of the THP‐1 to SA222, SA333, SA‐L1, and SA‐L2 at MOI 50 for 1 h. LDH cytotoxicity assay was performed using a Cytotoxicity Detection Kit (Promega, Australia). Briefly, the supernatants were centrifuged at 250×*g* at 4°C for 4 min and 50 µL aliquots were transferred into duplicate wells of a 96‐well plate. Then, 50 µL of Substrate Mix dissolved in Assay Buffer was added and the plate was then incubated for 30 min at room temperature, in the dark. Finally, the absorbance was read at 490 nm using an Imark Microplate Reader (Bio‐Rad Laboratories Inc, USA) following the addition of 50 µL Stop Solution. Cell viability percentage was reported after correction for the absorbance of the medium as background. Viability studies were carried out independently three times with three wells per bacteria.

### 4.10 Bioinformatics, Statistical Analysis, and Data Availability

Pairwise alignment and multiple‐sequence alignment between the genomes were performed using MAFFT, and the phylogenetic tree was inferred using PhyML tree in Geneious Prime (v2022.2.2). The ANI of assembled genomes was calculated using a FastANI web tool available at https://proksee.ca [66]. Statistical analysis was performed using Prism (v9.4) (GraphPad Software, USA), and the graphs were made in R (v4.2.0). The difference in means of biofilm and metabolic activity was tested using the Student *t*‐test. *p* < 0.05 was considered statistically significant unless stated otherwise. The genomic data (reads and assemblies), supplementary data, protocol, and materials pertaining to this research are also available in a public database under the following doi address: http://10.6084/m9.figshare.22696627.

## Author Contributions

Conceptualization: S.V. and R.N. Methodology, formal analysis, and investigation: R.N., G.H., G.B., M.R., G.S., and S.F. Data curation: G.H. and G.B. Writing—original draft: R.N. Writing—review and editing: G.H., G.B., S.F., M.R., G.S., K.S., A.J.P., P.J.W., and S.V. Visualization: R.N., G.H., and G.B. Supervision: S.V., P.J.W., and A.J.P. Funding acquisition: S.V. and P.J.W. All authors have read and approved the final manuscript.

## Conflicts of Interest

The authors declare no conflicts of interest.

## Ethics Statement

Ethics approval for the use of clinical isolates (CIs) and primary HNEpCs was obtained from the Human Research Ethics Committee of the Central Adelaide Local Health Network (HREC/18/CALHN/69). No animals were used in this study.

## Supporting information




**Figure S1**: Identification of prophage region of SA222, SA333 and SA‐L1 by PHASTEST. (A) S. aureus SA222 only harbours one intact prophage region (52.5 kb). (B) S. aureus SA333 harbours two intact prophage regions (50.8 kb and 43.8 kb). It is noted that the first prophage in S. aureus SA333 is almost like the one from S. aureus SA222 but has two transposases integrated into the prophage region (red box).(C) Laboratory‐generated S. aureus SA‐L1 harbours two intact prophages, one from S. aureus SA222 and one from S. aureus SA333. The second prophage was induced from S. aureus SA333 and inserted into S. aureus SA222. (D) The genetic mapping of jSa2int prophage from S. aureus SA333. Note the gain of two transposase enzymes compared to the same prophage present in S. aureus SA222.
**Figure S2**: Chromosomal location jSa3int prophage and correlations between SA222, SA333 and SA‐L1. (A) Chromosomal location of the jSa3int prophage insert in SA‐L1. (B) A heatmap representing the genomic similarities between donor (SA333), recipient (SA222) and laboratory‐generated double lysogen (SA‐L1 and SA‐L2). The numbers inside the square represent aligned identical bases/nucleotides between the strains in percentage. (C) Principal component analysis (PCA) of proteomics (triplicates) between SA222, SA333 and SA‐L1. The analysis shows that the proteomics of triplicates clustered together, indicating consistency of the secretome. (D) Pearson's correlation of proteomics (triplicates) between SA222, SA333 and SA‐L1 also represents consistency in release factors in the secretome.
**Figure S3**: A phylogenetic tree (relatedness) of all the isolates used for host‐range testing, their disease type, prophage distribution, sequence type (ST) and sensitivity towards jSa2int and jSa3int prophages. There was no correlation between sequence type (ST) and phage sensitivity when the isolates were tested against jSa2int and jSa3int prophages released from SA222 and SA333 respectively. However, it is noted that the released (pro)phages from both isolates could not infect clinical isolates that had similar (jSa2int and jSa3int) prophages as resident prophages.
**Figure S4**: Comparative cell adhesion of RN4220, SA222, SA333 and lysogens SA‐L1 and SA‐L2. There was no significant difference in adhesion of lysogens (SA‐L1 & SA‐L2) compared to its recipient bacteria (SA222) to primary human nasal epithelial cells. ns = not significant.
**Table S1**: Total colony forming unit (CFU) of supernatant and cell lysate.

## Data Availability

All the data generated in this study are included in the manuscript and is available as a supplementary information. All the supplementary data, protocol, and materials pertaining to this research are available in a public database under the following doi address: http://10.6084/m9.figshare.22696627. Raw data (gDNA, proteomics, and the codes) are publicly available via GitHub and can be downloaded from: https://github.com/gbouras13/Roshan_S_Aureus_Proteomics_Comp_Genomics. The raw genomic data (reads and assemblies) can be found in the NCBI Sequence Read Archive (SRA) under project number PRJNA914892, specifically with sample numbers SAMN32360844 (C222) and SAMN32360890 (C333). Clinical data, if required, can be obtained by request to the corresponding author S.V. This manuscript is part of a PhD research thesis entitled “Role of prophages in *Staphylococcus aureus* virulence and pathogenicity” and certain portion of the research is publicly available at the university repository [67] and bioRxiv preprint server [68].
