# Supplementary material for: A φSa3int (NM3) Prophage Domestication in Staphylococcus aureus Leads to Increased Virulence Through Human Immune Evasion
Source: MedComm (2020). 2025 Aug 8;6(8):e70313. doi: 10.1002/mco2.70313 (PMC12332420; doi:10.1002/mco2.70313)
Supplement: Supplementary file 1 — Figure S1: Identification of prophage region of SA222, SA333 and SA‐L1 by PHASTEST. (A) S. aureus SA222 only harbours one intact prophage region (52.5 kb). (B) S. aureus SA333 harbours two intact prophage regions (50.8 kb and 43.8 kb). It is noted that the first prophage in S. aureus SA333 is almost like the one from S. aureus SA222 but has two transposases integrated into the prophage region (red box).(C) Laboratory‐generated S. aureus SA‐L1 harbours two intact prophages, one from S. aureus SA222 and one from S. aureus SA333. The second prophage was induced from S. aureus SA333 and inserted into S. aureus SA222. (D) The genetic mapping of jSa2int prophage from S. aureus SA333. Note the gain of two transposase enzymes compared to the same prophage present in S. aureus SA222. Figure S2: Chromosomal location jSa3int prophage and correlations between SA222, SA333 and SA‐L1. (A) Chromosomal location of the jSa3int prophage insert in SA‐L1. (B) A heatmap representing the genomic similarities between donor (SA333), recipient (SA222) and laboratory‐generated double lysogen (SA‐L1 and SA‐L2). The numbers inside the square represent aligned identical bases/nucleotides between the strains in percentage. (C) Principal component analysis (PCA) of proteomics (triplicates) between SA222, SA333 and SA‐L1. The analysis shows that the proteomics of triplicates clustered together, indicating consistency of the secretome. (D) Pearson's correlation of proteomics (triplicates) between SA222, SA333 and SA‐L1 also represents consistency in release factors in the secretome. Figure S3: A phylogenetic tree (relatedness) of all the isolates used for host‐range testing, their disease type, prophage distribution, sequence type (ST) and sensitivity towards jSa2int and jSa3int prophages. There was no correlation between sequence type (ST) and phage sensitivity when the isolates were tested against jSa2int and jSa3int prophages released from SA222 and SA333 respectively. However, it is noted that [file MCO2-6-e70313-s001.pdf]

SUPPLEMENTARY MATERIALS & SUPPLEMENTARY METHODS

A φSa3int (NM3) prophage domestication in *Staphylococcus aureus* leads to increased virulence through human immune evasion

Running title:

Sa3int prophage enhances *Staphylococcus aureus* virulence

Authors:

Roshan Nepal[1,2,3], Ghais Houtak[1,2], George Bouras[1,2], Sholeh Feizi[1,2], Gohar Shaghayegh[1,2], Keith Shearwin[4], Mahnaz Ramezanpour[1,2], Alkis James Psaltis[1,2], Peter-John Wormald[1,2], Sarah Vreugde[1,2]*

[1] The Faculty of Health and Medical Sciences, The University of Adelaide, Adelaide, Australia.

[2] The Department of Surgery-Otolaryngology Head and Neck Surgery, The Basil Hetzel Institute for Translational Health Research, Central Adelaide Local Health Network, South Australia, Australia.

[3] Commonwealth Scientific and Industrial Research Organisation (CSIRO), Agriculture and Food, Livestock and Aquaculture, Hobart, TAS, Australia

[4] School of Biological Sciences, Faculty of Sciences, Engineering and Technology, The University of Adelaide, Adelaide, Australia.

*** Correspondence:**

Sarah.Vreugde@adelaide.edu.au

SUPPLEMENTARY FIGURE(S)

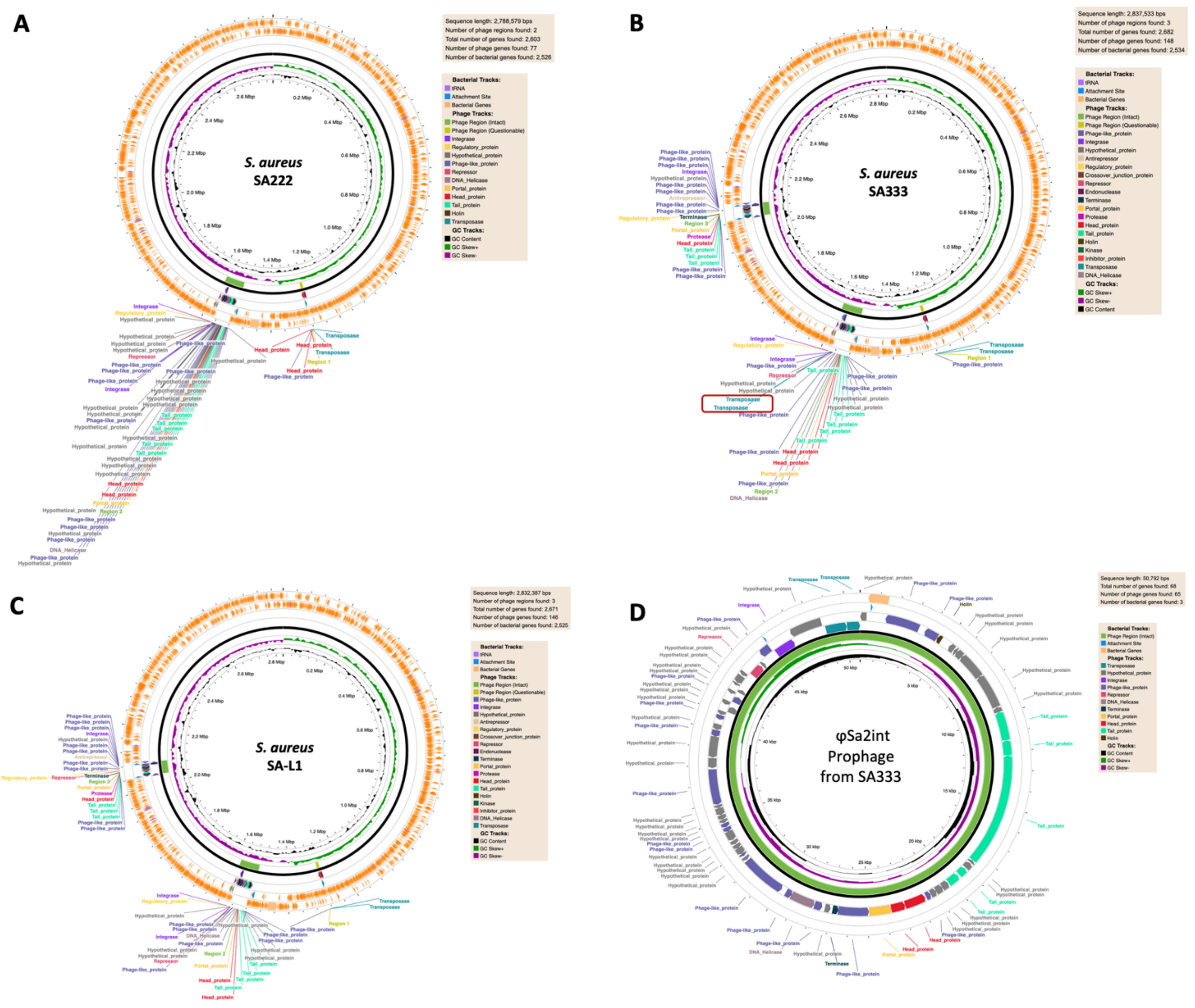

Figure S1 | Identification of prophage region of SA222, SA333 and SA-L1 by PHASTEST.

(A) *S. aureus* SA222 only harbours one intact prophage region (52.5 kb).

(B) *S. aureus* SA333 harbours two intact prophage regions (50.8 kb and 43.8 kb). It is noted that the first prophage in *S. aureus* SA333 is almost like the one from *S. aureus* SA222 but has two transposases integrated into the prophage region (red box).

(C) Laboratory-generated *S. aureus* SA-L1 harbours two intact prophages, one from *S. aureus* SA222 and one from *S. aureus* SA333. The second prophage was induced from *S. aureus* SA333 and inserted into *S. aureus* SA222.

(D) The genetic mapping of φSa2int prophage from *S. aureus* SA333. Note the gain of two transposase enzymes compared to the same prophage present in *S. aureus* SA222.

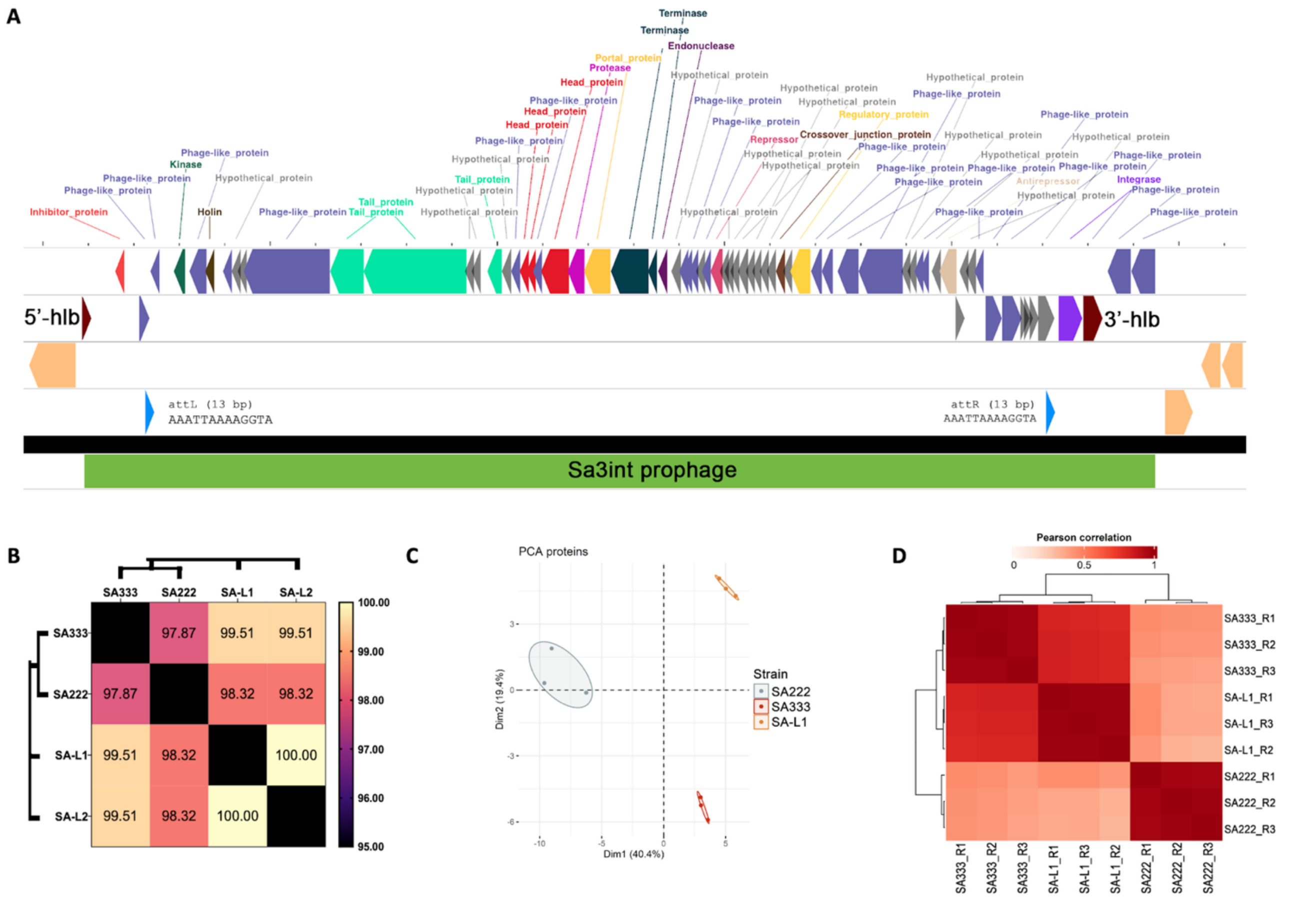

Figure S2 | Chromosomal location φSa3int prophage and correlations between SA222, SA333 and SA-L1.
(A) Chromosomal location of the φSa3int prophage insert in SA-L1.
(B) A heatmap representing the genomic similarities between donor (SA333), recipient (SA222) and laboratory-generated double lysogen (SA-L1 and SA-L2). The numbers inside the square represent aligned identical bases/nucleotides between the strains in percentage.
(C) Principal component analysis (PCA) of proteomics (triplicates) between SA222, SA333 and SA-L1. The analysis shows that the proteomics of triplicates clustered together, indicating consistency of the secretome.
(D) Pearson's correlation of proteomics (triplicates) between SA222, SA333 and SA-L1 also represents consistency in release factors in the secretome.

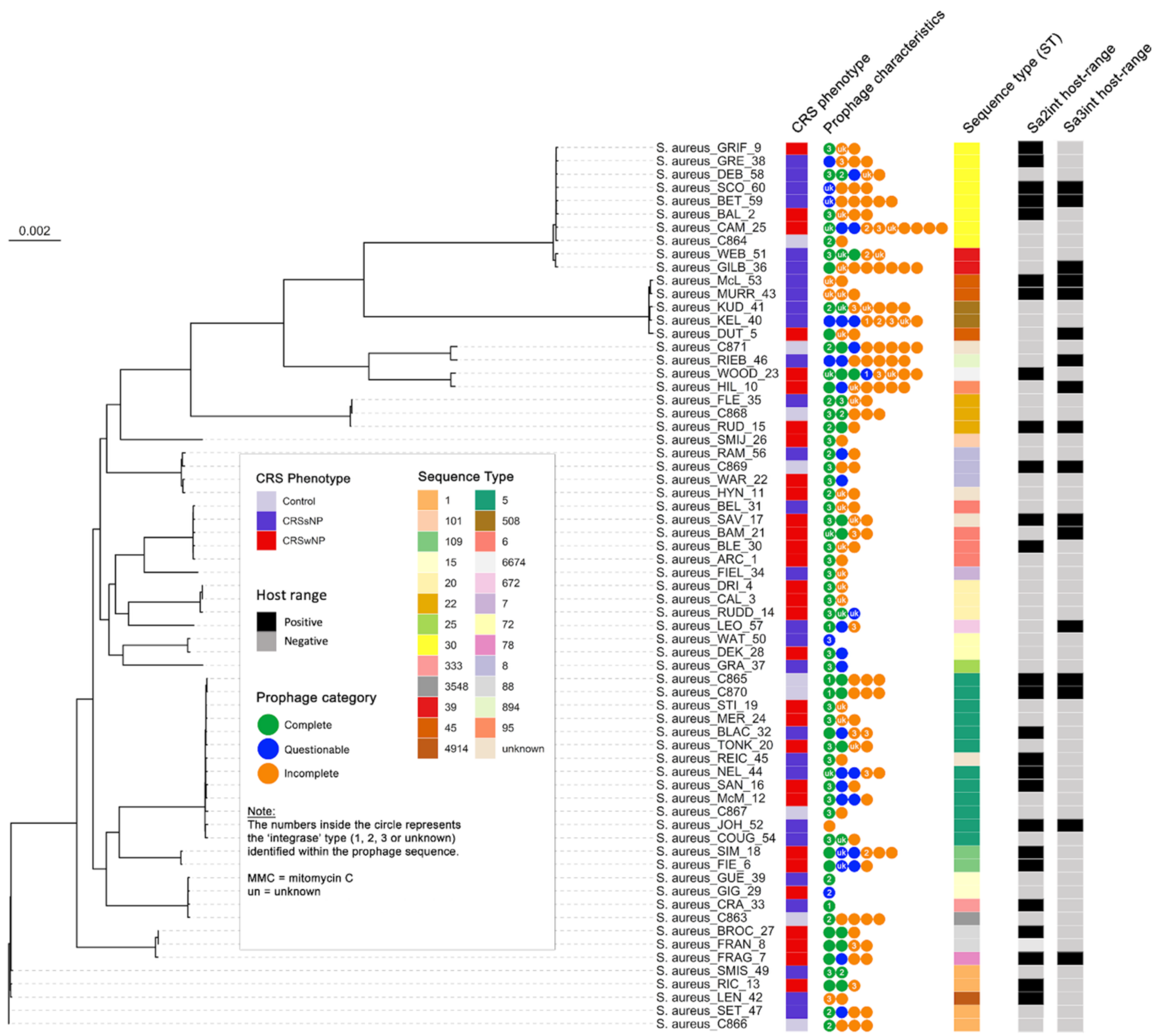

Figure S3 | A phylogenetic tree (relatedness) of all the isolates used for host-range testing, their disease type, prophage distribution, sequence type (ST) and sensitivity towards φSa2int and φSa3int prophages. There was no correlation between sequence type (ST) and phage sensitivity when the isolates were tested against φSa2int and φSa3int prophages released from SA222 and SA333 respectively. However, it is noted that the released (pro)phages from both isolates could not infect clinical isolates that had similar (φSa2int and φSa3int) prophages as resident prophages.

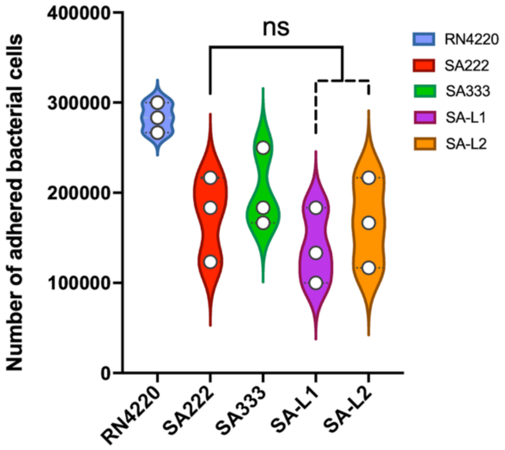

Figure S4 | Comparative cell adhesion of RN4220, SA222, SA333 and lysogens SA-L1 and SA-L2.
There was no significant difference in adhesion of lysogens (SA-L1 & SA-L2) compared to its recipient bacteria (SA222) to primary human nasal epithelial cells. ns = not significant

SUPPLEMENTARY TABLE(S)

Supplementary Table S1 | Total colony forming unit (CFU) of supernatant and cell lysate

Bacterial isolates	Supernatant (CFU/ml)	Cell lysate (CFU/ml)	Supernatant + Cell lysate (CFU/ml)
**SA222**	9.83E+05	1.88E+04	1.00E+06
	9.17E+05	3.40E+04	9.51E+05
	8.17E+05	2.60E+04	8.43E+05
	9.33E+05	3.68E+04	9.70E+05
	9.00E+05	3.25E+04	9.33E+05
	1.07E+06	3.65E+04	1.10E+06
**SA333**	9.33E+05	3.70E+04	9.70E+05
	9.50E+05	3.25E+04	9.83E+05
	1.10E+06	3.65E+04	1.14E+06
	9.50E+05	1.90E+04	9.69E+05
	8.83E+05	3.40E+04	9.17E+05
	8.33E+05	2.75E+04	8.61E+05
**SA-L1**	1.82E+06	2.30E+03	1.82E+06
	1.65E+06	3.70E+03	1.65E+06
	1.45E+06	3.80E+03	1.45E+06
	2.00E+06	2.32E+03	2.00E+06
	1.92E+06	3.35E+03	1.92E+06
	2.00E+06	3.17E+03	2.00E+06
**SA-L2**	2.05E+06	4.00E+02	2.05E+06
	1.88E+06	3.17E+02	1.88E+06
	1.98E+06	3.67E+02	1.98E+06
	1.77E+06	3.50E+02	1.77E+06
	1.48E+06	2.67E+02	1.48E+06
	1.50E+06	3.17E+02	1.50E+06
**SA222 in media**	1.08E+06		1.08E+06
	1.32E+06		1.32E+06
	1.08E+06		1.08E+06
	1.18E+06		1.18E+06
	1.25E+06		1.25E+06
	1.17E+06		1.17E+06
**SA-L1 in media**	1.43E+06		1.43E+06
	1.75E+06		1.75E+06
	1.43E+06		1.43E+06
	2.00E+06		2.00E+06
	9.00E+05		9.00E+05
	1.95E+06		1.95E+06

CFU = colony forming units. The experiments were performed in 3 biological replicates.

SUPPLEMENTARY METHODS

4.2 (Continued) | Genomic DNA extraction, sequencing, genome assembly and annotation

The genomic DNA (gDNA) of all *S. aureus* CIs were extracted using DNeasy Blood & Tissue Kit (Cat. #69504, Qiagen Pty. Ltd, Australia) according to the manufacturer's guidelines with slight modifications. Briefly, 700 µl of overnight broth culture in TSB was centrifuged (4000 x g) in a 1.5 ml Eppendorf tube for 10 minutes. The pellet was suspended in 180 µl of enzymatic lysis buffer (20 mM Tris-Cl, pH8; 2mM sodium EDTA; 1.2% Triton X-100, 200 µg/ml final concentration lysostaphin, filter sterilized) and incubated at 37°C for 30 minutes. Then 25 µl of proteinase K and 200 µl of Buffer AL (undiluted, provided with extraction kits) were added and vortexed followed by incubation at 56°C for 30 min. After 30 min, 200 µl of 99% ethanol (chilled) was added and vortexed to mix. The mixture was then transferred to DNeasy Mini Spin column (Qiagen Pty. Ltd, Australia, Cat. #69504), and DNA was extracted following the manufacturer's guidelines.

The extracted gDNA was sequenced using the short-read Illumina platform (Illumina Inc, San Diego, USA) and in-house long-read Oxford Nanopore Technology (ONT) using the MinION Mk1C device (Oxford Nanopore Technologies, Oxford, UK) following the manufacturer's instructions and in-house established protocol [1]. Briefly, the short-read sequencing was done on Illumina NextSeq 550 platform using NextSeq 500/550 Mid-Output kit (v2.5) (Illumina Inc, San Diego, USA) at a commercial sequencing facility SA Pathology (Adelaide, SA, Australia). Briefly, gDNA was isolated using the NucleoSpin Microbial DNA kit (Machery-Nagel GmbH, Duren, Germany). Sequencing libraries were prepared using a modified protocol for the Nextera XT DNA library preparation kit (Illumina Inc, San Diego, USA). The gDNA was fragmented and amplified using a low-cycle PCR reaction. After the manual purification and normalisation of the amplicon library, 150 bp reads were obtained. Long-read whole genome sequencing was performed using MinION flowcells (R9.4.1) with the Rapid Barcoding Kit (Oxford Nanopore Technology, UK, #Cat: SQK-RBK 110.96) according to the manufacturer's instructions. In brief, 50 ng of gDNA from each clinical isolate was used for sequencing. Base-calling was conducted with Guppy (v6.2.11) (mode = super accuracy) using the 'dna_r9.4.1_450bps_sup.cfg' configuration (Oxford Nanopore Technologies, UK). Chromosome Assemblies: Complete chromosomal *Staphylococcus aureus (S. aureus)* assemblies were created using a customised Snakemake pipeline [2] which can be found at https://github.com/gbouras13/Nanopore_Bacterial_Assembly_Pipeline, also available as a Snaketool enabled command line tool *hybracter* [3]. Long reads were subsampled to generate a 250 Mbp read set for each sample using Rasusa [4]. Adapters and barcodes were trimmed using Porechop v0.2.4 [5]. Short reads were filtered, with low-quality regions and adapters trimmed using fastp [6]. Long-read-only assemblies were created using Flye (v2.9.1) with '--nano-hq' specified [7]. Assemblies, including contigs with a length greater than 2.5 Mbp, were kept and denoted as the putative chromosomal. The resulting chromosomes were polished with long reads first using Medaka (v1.7.0) (Oxford Nanopore Technologies, 2022), then with short reads using Polypolish (v0.5.0) [8]. After the first round of polishing, the chromosomes were reoriented to begin at the putative *dnaA* gene using the customised python program dnaapler [9]. Chromosomes were then polished a second time with Polypolish, followed by POLCA [10]. These reads were then assembled using Unicycler (v0.5.0) [11] to generate final plasmid contigs. Genomic analysis: Chromosomes were

annotated with Bakta (v0.5.0) [12]. All clinical isolates (Cis) were typed (clonal complex) according to the PubMLST database using MLST [13, 14]. Antimicrobial resistance and virulence genes were identified by screening all isolate contigs through the Comprehensive Antibiotic Resistance Database [15] and Virulence Factor Database [16] using ABRicate (v1.0.1) [17]. To detect structural changes such as repeats, large deletions, or where insertions have been introduced, we used NucDiff (v2.0) [18]. We used Snippy to detect single nucleotide polymorphisms (SNPs) between Cis [19]. Dot plots were generated using D-GENIES (v1.4) performing large genome alignments using minimap2 [20].

4.6 (Continued) | Growth curve, biofilm biomass, biofilm metabolic activity and adhesion assay of *S. aureus* to primary human nasal epithelial cells (NHEpC)

Bacterial growth kinetics were determined by measuring the optical density of broth culture at 600 nm (OD600). Briefly, 100 µl of 1.0 McFarland standard unit (MFU in saline, prepared from overnight cultured colonies on NA plates) was added to 15.0 ml of TSB in a 50 ml Falcon tube. The tubes were incubated at 37°C in a shaking incubator (180 rpm). Every hour, 100 µl of culture was removed and mixed with 900 µl of sterile TSB in a cuvette. The OD600 was then measured using a SmartSpec™ 3000 UV/Vis spectrophotometer (Bio-Rad Laboratories Inc, California, USA).

The biofilm variation between the clinical isolates and lysogens was qualitatively assessed by culturing the bacteria on modified Congo red agar (CRA) (37 gm/l brain heart infusion broth supplemented with 50 g/l sucrose, 0.8 g/l Congo red stain and 1.0 % agar) according to Freeman et al.[21]. Colony morphology of SA222, SA333 and SA-L1/SA-L2 on CRA was assessed after 48 h of incubation at 37°C. Further, biofilm biomass and biofilm metabolic activity (cell viability) was performed using microtiter crystal violet (CV) assay and alamarBlue® cell viability assay respectively as per manufacturer's guidelines (Life Technologies, Oregon, USA) in biofilms established for 48 hours in 96-well flat-bottomed (Costar, Corning Incorporated, USA. #Ref: 3599) and 96-well flat and clear bottom black assay plate (Costar, Corning Incorporated, USA. #Ref: 3603) respectively, as described earlier [22]. Briefly, the overnight NA culture of *S. aureus* was adjusted to 1.0 McFarland standard and diluted 1:15 in tryptic soy broth. One-hundred-fifty microliters of each diluted culture were pipetted into the inner wells of the respective 96-well plates. The peripheral wells were filled with sterile water, sealed with aluminium foil and incubated at 37°C in an orbital shaker (80 rpm). After 48 h of incubation, planktonic cells were carefully aspirated, and the plates were washed twice with phosphate buffered saline (PBS, 1X) followed by the crystal violet or alamarBlue® assay. For CV, plates were air-dried and 180 µl of 0.01% CV solution was added to each inner well and left at room temperature for staining. After 10 minutes, the excess CV was aspirated, washed twice with PBS (1X) and air-dried. Finally, the biomass-bound crystal violet was solubilized in 200 µl of 30% acetic acid. The biomass was measured in terms of absorbance (OD_{600}) at 600 nm using CLARIOstar Plus (BMG Labtech, Ortenberg, Germany). Similarly, for biofilm metabolic activity, 200 µl working solution of alamarBlue® (1X) was added to each inner well, covered with aluminium foil and incubated at 37°C in an orbital shaker (80 rpm). Resorufin fluorescence was monitored at the 1 h interval for up to 6 h using the CLARIOstar Plus (BMG Labtech, Ortenberg, Germany) microplate reader (excitation = 530 nm, emission = 590

nm). The metabolic activity was expressed as fluorescence units. All experiments were performed in triplicate with six technical replicates.

The adhesion of *S. aureus* clinical strains and lysogens to primary human nasal epithelial cells (HNEpC) was studied following the protocol by Yang and Ji with slight modifications [23]. Briefly, primary HNEpCs were cultured in RPMI 1640 working media (supplemented with 10% FBS and 1% antibiotic-antimycotic, hereafter RPMI 1640-WM) to 70% confluency in a tissue culture flask (T-75, Sarstedt, Nümbrecht, Germany) at 37°C, 5% CO_2 incubator and transferred (1 ml/well) into a 24-well tissue culture plate (Sarstedt, Nümbrecht Germany). An overnight broth culture of bacteria was made in 5 ml of TSB, pelleted and resuspended and the bacterial density was then adjusted to ~0.3 OD600. In a separate tube, 5.0 ml of RPMI 1640+10% FBS (without antibiotics) was aliquoted, and 150 μl of the diluted bacteria (OD600 = 0.3) was added to prepare the working bacterial culture. The cell culture media was replaced with RPMI 1640+10% FBS media with and without bacterial suspensions and cells incubated for 2 h at 37°C, 5% CO_2 incubator. The wells were again washed and then incubated with 400 μl of 0.025% Triton X-100 by pipetting, transferred into corresponding Eppendorf tubes and mixed by vortexing for 30 s. The recovered bacteria were serially diluted in sterile PBS (up to 10^{-4}), and 20 μl was spotted in TSA for CFU estimation. The plates were dried and incubated at 37°C along with previous plates containing serially diluted working bacterial culture spots. The next day, the colonies in each plate were counted. The relative adhesion was calculated using the following formulae: Relative adhesion = $\frac{Adhesion\ of\ the\ lysogen}{Adhesion\ of\ the\ parent\ strain} x\ 100\%$

4.7 (Continued) | Downstream analysis of the secretome (digestion and proteomics analysis)

The proteomics of the secretome was analyzed using a data-independent acquisition mass spectrometry (DIA-MS) using Orbitrap Fusion Lumos Tribrid Mass Spectrometer (Thermo Fisher Scientific, USA) with Dionex UltiMate™ 3000 UHPLC system (Thermo Fisher Scientific, USA) at The Flinders Omics Facility, Flinders University according to established protocol (Supplementary method 5.7). Briefly, a few colonies from overnight cultured nutrient agar plates were dissolved in sterile saline to obtain 1.0 MFU standard. One hundred microliters of the solution were added to 15.0 ml of TSB in a 50.0 ml Falcon tube and incubated at 37°C in a shaking incubator (180 rpm, 45° angle). After ~7 hours, the tube was briefly vortexed and centrifuged at 4000 x g for 10 min. The secretome was sterilized by passing it through a 0.2 μm syringe filter (25 mm, Acordisc®, Pall International, Fribourg, Switzerland) and concentrated using a Pierce™ Protein Concentrator PES (3K MWCO, #Cat: 88525, Thermo Scientific, USA) by centrifuging the sample down to approximately 2 ml. The protein concentration was determined using NanoOrange™ Protein Quantitation Kit (Invitrogen, USA. #Cat: N6666) as per the manufacturer's instruction. The proteins were then processed at the Flinders Omics Facility, Flinders University.

The proteins were reduced with tris(2-carboxyethyl)phosphine (TCEP, 10 mM, 30 min, 56°C) and alkylated in the dark with chloroacetamide (20 mM, 30 min, RT) (Sigma-Aldrich, Darmstadt, Germany). The reduced proteins were washed using Sera-

Mag™ carboxylate-modified magnetic beads (Cytiva, USA) following the manufacturer's instructions. The proteins were then digested using trypsin (1:20 enzyme-to-substrate ratio) (Promega, Madison, USA) and incubated overnight at 37°C. The proteins were then bound to Sera-Mag™ carboxylate-modified magnetic beads (Cytiva, USA) by adding acetonitrile (final concentration of ≥ 95%). The sample was resuspended in 0.1% formic acid (Buffer A) to achieve a final peptide concentration of 1 µg/3 µl) before mass spectrometry acquisition. Aliquots from five replicates were pooled together to ensure the presence of all peptides and the preparation of the chromatogram library.

Liquid chromatography: The digested peptides were then analyzed using a data-independent acquisition mass spectrometry (DIA-MS) using Orbitrap Exploris™ 480 Mass Spectrometer (Thermo Fisher Scientific, USA) with Dionex UltiMate™ 3000 RSnano UHPLC system (Thermo Fisher Scientific, USA) at The Flinders Omics Facility, Flinders University according to established protocol. Digested protein sample (1.0 µg) was injected into an in-house 75 µm (inner diameter) trap column with fused silica capillary ReproSil-Pur 120 C18-AQ beads (1.9 µm, 120 Å, Dr. Maisch, Ammerbuch, Germany) to 25 cm, coupled with a PepMap™ 100 trap cartridge (0.3 x 5 mm, 5 µm C18, Thermo Fisher Scientific, USA). For each injection, 1 µg of peptides was loaded and separated using a 120-min gradient from 3-31.2% solvent B (0.1% formic acid in 80% acetonitrile), followed by a 30-minute washing and equilibration gradient. Solvent A was 0.1% formic acid in water.

Library generation and quantitative shotgun data-independent acquisition mass spectrometry (DIA-MS): A pooled sample comprised of 1.5 µl of protein digest was used to generate a sample project-specific spectral library for data-dependent analysis (DDA). Six gas phase fractionation (GPF) chromatogram library acquisitions, each spanning a narrow m/z range across the 350-1200 m/z total mass range (350-500 m/z method 1, 490-610 m/z method 2, 600-710 m/z method 3, 700-810 m/z method 4, 800-910 m/z method 5, 900-1200 m/z method 6) were used. For each DDA-GPF analysis, 2.0 µl of the pooled sample was used with a 3-second cycle time instrument method. Briefly, a narrow spectra (MS1) scan matching one of the six m/z mass ranges was performed using an Orbitrap resolution of 60,000. A normalised AGC target of 3e6 with an auto maximum injection time mode was used. An intensity threshold of 2.5e5 and dynamic exclusion time of 45 seconds was employed for all data-dependent second stage (MS2) scans that were acquired at 15,000 resolution, AGC target 5e4, 33% normalised collision energy (NCE) in the HCD cell, with an auto maximum inject time mode.

For the DIA runs, the Orbitrap Exploris™ 480 Mass Spectrometer (Thermo Fisher Scientific, USA) was configured to acquire 37 16 m/z precursor isolation windows (396.43-1004.70 m/z), followed by 37 16 m/z windows (400.43-1008.70 m/z) creating a staggered window pattern. An MS2 resolution of 15,000, AGC target 5e4, maximum inject time of 20 ms, and normalised HDC collision energy of 28 were employed for all DIA scans. Precursor spectra over a 390-1010 m/z mass range were acquired prior to DIA scans with a resolution of 60,000, AGC target 3e5, and maximum inject time 100 ms were used for all full scan MS spectra.

Bibliography

1. Shaghayegh, G., et al., *Chronic rhinosinusitis patients display an aberrant immune cell localization with enhanced S aureus biofilm metabolic activity and biomass.* Journal of Allergy and Clinical Immunology, 2023. **151**(3): p. 723-736.e16.
2. Mölder, F., et al., *Sustainable data analysis with Snakemake.* F1000Research, 2021. **10**: p. 33.
3. Bouras, G. *hybracter* hybracter; Available from: https://github.com/gbouras13/hybracter.
4. Hall, M., *Rasusa: Randomly subsample sequencing reads to a specified coverage.* Journal of Open Source Software, 2022. **7**(69): p. 3941.
5. Wick, R.R. *Porechop*. Porechop; Available from: https://github.com/rrwick/Porechop.
6. Chen, S., et al., *fastp: an ultra-fast all-in-one FASTQ preprocessor.* Bioinformatics, 2018. **34**(17): p. i884-i890.
7. Kolmogorov, M., et al., *Assembly of long, error-prone reads using repeat graphs.* Nature Biotechnology, 2019. **37**(5): p. 540-546.
8. Wick, R.R. and K.E. Holt, *Polypolish: Short-read polishing of long-read bacterial genome assemblies.* PLOS Computational Biology, 2022. **18**(1): p. e1009802.
9. Bouras, G. *dnaapler*. dnaapler; Available from: https://github.com/gbouras13/dnaapler.
10. Zimin, A.V. and S.L. Salzberg, *The genome polishing tool POLCA makes fast and accurate corrections in genome assemblies.* PLOS Computational Biology, 2020. **16**(6): p. e1007981.
11. Wick, R.R., et al., *Unicycler: Resolving bacterial genome assemblies from short and long sequencing reads.* PLoS Comput Biol, 2017. **13**(6): p. e1005595.
12. Schwengers, O., et al., *Bakta: rapid and standardized annotation of bacterial genomes via alignment-free sequence identification.* Microb Genom, 2021. **7**(11).
13. Seemann, T. *mlst*. mlst; Available from: https://github.com/tseemann/mlst.
14. Jolley, K.A., J.E. Bray, and M.C.J. Maiden, *Open-access bacterial population genomics: BIGSdb software, the PubMLST.org website and their applications.* Wellcome Open Res, 2018. **3**: p. 124.
15. Jia, B., et al., *CARD 2017: expansion and model-centric curation of the comprehensive antibiotic resistance database.* Nucleic Acids Res, 2017. **45**(D1): p. D566-D573.
16. Liu, B., et al., *VFDB 2019: a comparative pathogenomic platform with an interactive web interface.* Nucleic Acids Res, 2019. **47**(D1): p. D687-D692.
17. Seemann, T. *Abricate*. Abricate; Available from: https://github.com/tseemann/abricate.
18. Khelik, K., et al., *NucDiff: in-depth characterization and annotation of differences between two sets of DNA sequences.* BMC Bioinformatics, 2017. **18**(1): p. 338.
19. Seemann, T. *Snippy: fast bacterial variant calling from NGS reads* 2015; Available from: https://github.com/tseemann/snippy.
20. Cabanettes, F. and C. Klopp, *D-GENIES: dot plot large genomes in an interactive, efficient and simple way.* PeerJ, 2018. **6**: p. e4958.
21. Freeman, D.J., F.R. Falkiner, and C.T. Keane, *New method for detecting slime production by coagulase negative staphylococci.* J Clin Pathol, 1989. **42**(8): p. 872-4.
22. Shaghayegh, G., et al., *Staphylococcus aureus biofilm properties and chronic rhinosinusitis severity scores correlate positively with total CD4+ T-cell frequencies and inversely with its Th1, Th17 and regulatory cell frequencies.* Immunology, 2023. **170**(1): p. 120-133.

23. Yang, J. and Y. Ji, *Investigation of Staphylococcus aureus Adhesion and Invasion of Host Cells*, in *Methicillin-Resistant Staphylococcus Aureus (MRSA) Protocols*, Y. Ji, Editor. 2014, Humana Press: Totowa, NJ. p. 187-194.
